# The progress of novel strategies on immune-based therapy in relapsed or refractory diffuse large B-cell lymphoma

**DOI:** 10.1186/s40164-023-00432-z

**Published:** 2023-08-14

**Authors:** Tingxun Lu, Jie Zhang, Zijun Y. Xu-Monette, Ken H. Young

**Affiliations:** 1 Department of Oncology, Affiliated Hospital of Jiangnan University, Wuxi, Jiangsu Province 214122 China; 2 Division of Hematopathology, Department of Pathology, Duke University School of Medicine, Durham, NC 27710 USA; 3 Duke Cancer Institute, Durham, NC 27710 USA

**Keywords:** Diffuse large B-cell lymphoma, Immunotherapy, Radiotherapy, Refractory, Relapsed

## Abstract

Diffuse large B-cell lymphoma (DLBCL) can be cured with standard front-line immunochemotherapy, whereas nearly 30–40% of patients experience refractory or relapse. For several decades, the standard treatment strategy for fit relapsed/refractory (R/R) DLBCL patients has been high-dose chemotherapy followed by autologous hematopoietic stem cell transplant (auto-SCT). However, the patients who failed in salvage treatment or those ineligible for subsequent auto-SCT have dismal outcomes. Several immune-based therapies have been developed, including monoclonal antibodies, antibody–drug conjugates, bispecific T-cell engaging antibodies, chimeric antigen receptor T-cells, immune checkpoint inhibitors, and novel small molecules. Meanwhile, allogeneic SCT and radiotherapy are still necessary for disease control for fit patients with certain conditions. In this review, to expand clinical treatment options, we summarize the recent progress of immune-related therapies and prospect the future indirections in patients with R/R DLBCL.

## Background

Diffuse large B-cell lymphoma (DLBCL) is the most common subtype of lymphoma, which accounts for approximately 40% of non-Hodgkin lymphoma (NHL) [1]. The most common standard first-line treatment remains R-CHOP regimens, mostly rituximab plus chemotherapy (cyclophosphamide, doxorubicin, vincristine, and prednisone) [2]. Approximately 60–70% of patients with DLBCL are cured with upfront therapy. However, 10 to 15% of patients exhibit primary refractory disease, and 20 to 25% of cases experience a relapse after the initial response [3]. The overall response rate (ORR) of relapsed or refractory (R/R) DLBCL treated with second-line therapy was 26%, and the median overall survival (OS) was 6.3 months [4]. Only about 50% of durable remissions were reached in R/R DLBCL patients who receive high-dose chemotherapy followed by autologous stem cell transplantation (auto-SCT) [5]. Patients not cured with auto-SCT or ineligible to auto-SCT or refractory to salvage chemotherapy may be considered for Chimeric Antigen Receptor (CAR) T cell therapy targeting CD19 [5]. Although auto-SCT and CAR-T cell therapy offer patients an opportunity for durable remission, many patients may not be eligible for auto-SCT or CAR-T cell therapy or relapse after these treatments [6]. In the last decade, the investigation of novel antigens, which can be targeted by immunotherapy and identified to eliminate malignant cells regardless of their molecular pathogenesis, has been constantly pursued. A variety of novel immunotherapies, including monoclonal antibodies (mAbs), antibody–drug conjugates (ADCs), bispecific antibodies (BsAbs), CAR-T cell therapies, immune checkpoint inhibitors (ICIs), and small molecules targeting unique pathways and biological process have been investigated. Meanwhile, traditional curable solutions, both for whole or local, such as allogeneic stem cell transplant (allo-SCT) and radiotherapy, are indispensable for immunotherapy in patients with R/R DLBCL. This review summarizes the progress in immune-related therapies approved and recommended by international guidelines. Furthermore, we also conclude novel agents under investigation, which might assist alone or in combination in treating R/R DLBCLs.

### MAbs

#### Tafasitamab

**Fig. 1:**
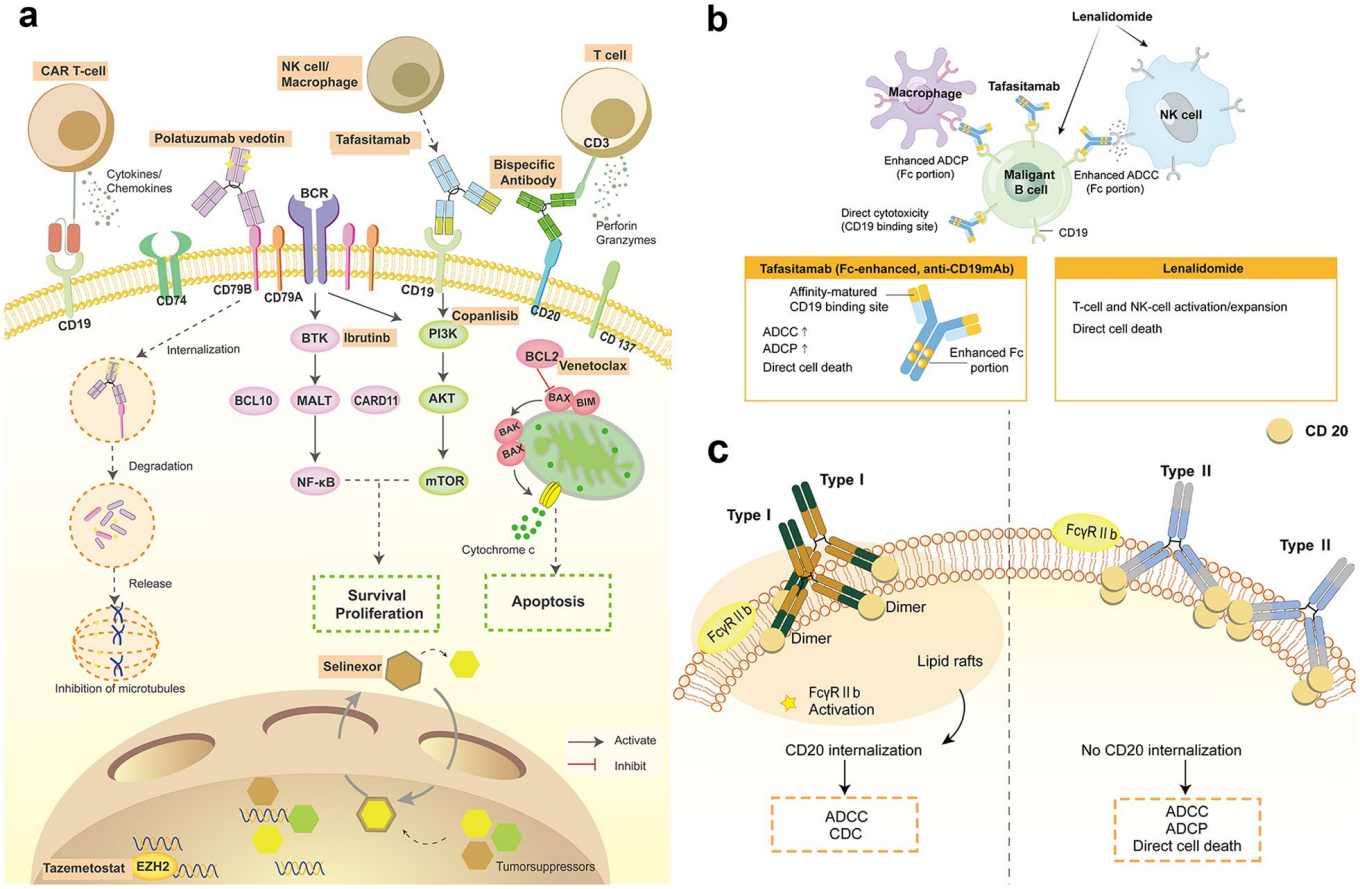
Monoclonal antibodies applied in R/R DLBCLs. Many monoclonal antibodies can be used in R/R DLBCLs. Among these, Tafasitamab showed an apparent synergistic effect with Lenalidomide (**a**). Tafasitamab shows direct cytotoxicity, ADCC and ADCP. Lenalidomide shows direct cytotoxicity, enhances ADCC and stimulates interferon-ϒ secretion, lowering the NK cell activation threshold and increasing NK cell proliferation by promoting interleukin-2 production (**b**). Obinutuzumab is a type II anti-CD20 monoclonal antibody with no CD20 internalization and a stronger antitumor effect than Rituximab (type I anti-CD20 monoclonal antibody) (**c**). *ADCC* antibody-dependent cell mediated cytotoxicity, *ADCP* antibody-dependent cell-mediated phagocytosis, *CDC* complement dependent cytotoxicity

CD19 is broadly and homogeneously expressed across B-cell malignancy, enhancing B-cell receptor signaling and tumor cell proliferation (Fig. 1a) [7, 8]. Tafasitamab (MOR208), an Fc-enhanced, humanized mAb [9], was well tolerated and showed encouraging efficacy in patients with R/R B-cell malignancy [10]. Based on preclinical research suggested that tafasitamab might have a synergistic effect with lenalidomide (Fig. 1b) [11]. A phase II multicentre, open-label, single-arm study (L-MIND, NCT02399085) tested the efficacy and safety of the combination of tafasitamab and lenalidomide R/R DLBCL patients who were ineligible for auto-SCT [11]. At the last follow-up (data cutoff: Oct 30, 2020), the ORR was 57.5% (46/80) with 40% of complete response (CR) and 17.5% of partial response (PR), the median duration of response (DOR), median progression-free survival (PFS) and median OS were 43.9 months, 11.6 months and 33.5 months, respectively [12]. The ORR in patients with primary refractory, rituximab-refractory, and refractory to their last line of therapy were 53.3%, 54.8%, and 60%, respectively [12]. Treatment-emergent adverse events (TEAEs) of any grade occurred in all patients, including hematological events, such as neutropenia (49%), anemia (34%), thrombocytopenia (31%), leukopenia (14%), febrile neutropenia (12%), and non-hematological events (most were grade 1–2), such as rash, diarrhea, asthenia, peripheral oedema. It's worth noting that patients with advanced age or who were not suitable for auto-SCT were included in this study, which indicates the safety and tolerability of this combined therapy. However, L-MIND is a single-arm study which did not compare the efficacies with other second and later-line regimens. A recent study compared the effectiveness of L-MIND results with other systemic therapies (systemic therapies pooled, BR, and R-GemOx) recommended by NCCN/ESMO guidelines for treating patients with R/R DLBCL matched in RE-MIND2. Consistent and significantly improved outcomes with L-MIND clinical trial versus matched other systemic therapies (Table 1) [13]. Tafasitamab is being evaluated in combination with bendamustine in a randomized phase II/III trial compared with BR in R/R DLBCL (B-MIND, NCT02763319). Besides, a multicenter, double-blind, placebo-controlled, randomized phase III trial (frontMIND) was designed to compare the efficacy and safety of tafasitamab plus lenalidomide and R-CHOP versus R-CHOP in high-intermediate and high-risk patients with previously untreated DLBCL is ongoing (NCT04824092).

**Table 1:** Comparative analysis results for L-MIND compared with other systemic therapies

Efficacy	L-MIND	Systemic therapies pooled	L-MIND	BR	L-MIND	R-GemOx
	N = 76		N = 75		N = 74	
ORR n (%)	51 (67.1)	37 (48.7)	50 (66.7)	41 (54.7)	51 (68.9)	34 (45.9)
HR (95% CI)	18.42 (1.905–34.204)		12.00 (4.657–28.173)		22.91 (6.285–38.722)	
*P* value	0.0323		0.1810		0.0076	
CR n (%)	29 (38.2)	16 (21.1)	29 (38.7)	21 (28.0)	29 (39.2)	17 (23.0)
HR (95% CI)	17.11 (0.579–32.952)		10.67 (-5.987–26.891)		16.22 (-0.548–32.318)	
*P* value	0.0324		0.2252		0.050	
Median TTNT (mo)	12.5	6.3	12.1	6.9	12.5	5.7
HR (95% CI)	0.461 (0.314–0.676)		0.527 (0.357–0.780)		0.423 (0.289–0.619)	
*P* value	< 0.0001		0.0011		< 0.0001	
Median OS (mo)	34.1	11.6	31.6	9.9	31.6	11.0
HR (95% CI)	0.553 (0.358–0.855)		0.418 (0.272–0.644)		0.467 (0.305–0.714)	
*P* value	0.0068		< 0.0001		0.0003	
Median PFS (mo)	12.1	5.5	12.1	7.9	14.1	5.1
HR (95% CI)	0.424 (0.278–0.647)		0.527 (0.344–0.809)		0.433 (0.288–0.653)	
*P* value	< 0.0001		0.0028		< 0.0001	
Median DOR (mo)	26.1		6.6		26.1	

*BR* bendamustine + rituximab, *R-GemOx* rituximab + gemcitabine + oxaliplatin, *ORR* objective response rate, *CR* complete response, *HR* hazard ratio, *CI* confidence interval, *TTNT* time to next treatment, *mo* months, *OS* overall survival, *PFS* progression-free survival, *DOR* duration of response

#### Obinutuzumab

Obinutuzumab (GA101), a glycoengineered, type II, anti-CD20 mAb, was superior to rituximab in human DLBCL xenograft models (Fig. 1c). In the phase II GAUGUIN study, the best ORR was 32% in the 1600/800 mg arm (DLBCL, N = 15) and 27% in the 400/400 mg study arm (DLBCL, n = 10), including 20% (5/25) of rituximab-refractory patients [14]. GOYA was a randomized phase III trial that compared G-CHOP with R-CHOP in patients with de novo advanced-stage DLBCL [15]. In this study, 1418 DLBCL patients were randomized to receive GA101 plus CHOP (G-CHOP) or R-CHOP. After a median follow-up of 29 months and three years PFS rates analyzed by investigators were 70% in G-CHOP and 67% in R-CHOP, respectively [15]. There was no improvement of PFS in previously untreated DLBCL patients treated with G-CHOP, compared to R-CHOP. However, G-CHOP was associated with higher rates of adverse events, especially neutropenia and infection. One study (NCT02220842) evaluated the efficacy of the combination of atezolizumab (Atezo) and GA101 in R/R DLBCL. Although this combination was safe and tolerable, the ORR was only 17% [16]. A phase Ib study reported the result of GA101 with venetoclax (BCL2 inhibitor) and polatuzumab vedotin (Pola), with an ORR of 29% in R/R DLBCLs [17]. Some studies combined with GA101 were completed, but the results have not been reported yet (NCT02987400, NCT03276468). Biomarker analyses may help to define a future role for GA101 in DLBCLs. Although sponsors terminated many clinical trials with GA101 in R/R DLBCLs, there are still several studies combined with GA101 are ongoing, including combination with pembrolizumab (NCT03401853), ViPOR-P (NCT04739813), venetoclax plus lenalidomide (NCT02992522), ICE chemotherapy (NCT02393157).

#### ADCs

**Fig. 2:**
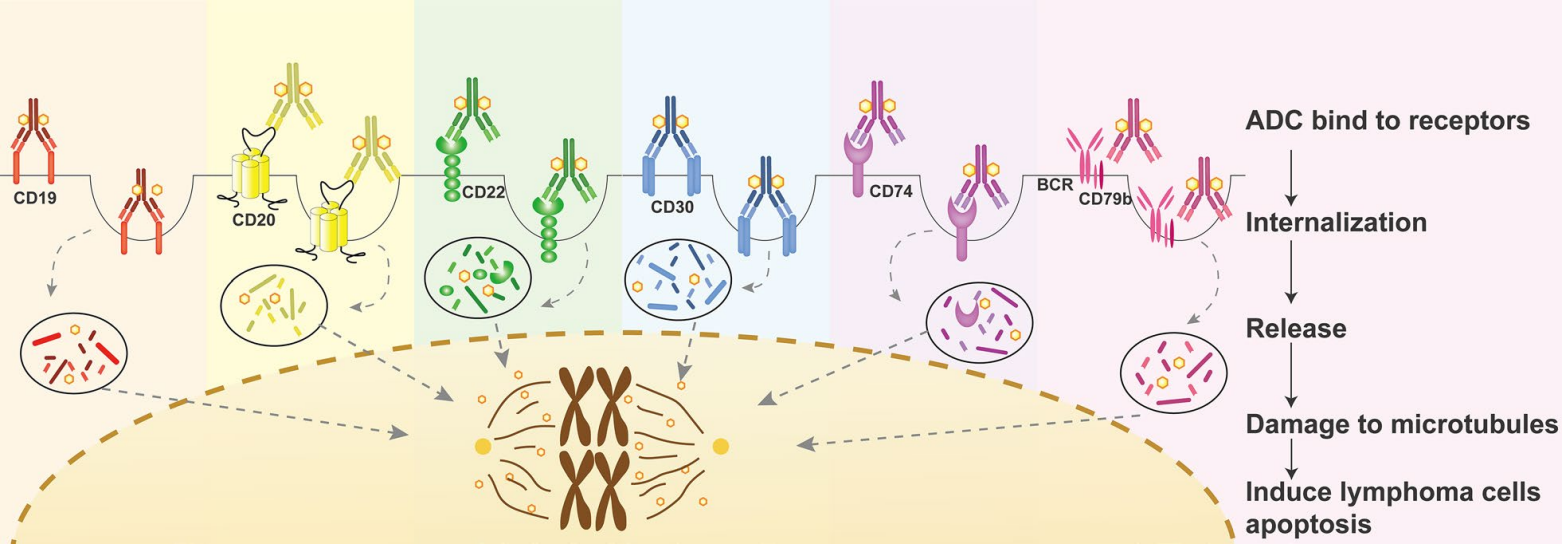
Antibody–drug conjugates used in R/R DLBCLs. This picture shows the mechanisms and processes of antibody–drug conjugates in lymphoma patients. Once the antibody binds the target antigen on the tumor cell surface. The complex is rapidly endocytosed and transported to lysosomes, where the effector molecule MMAE is released into the cytoplasm leading to cell toxicity. *ADC* antibody–drug conjugate

ADCs are biopharmaceutical compounds consisting of a cytotoxic agent linked to an antibody capable of targeted delivery of the payload to cells expressing the target protein. Unlike chemotherapy, ADCs are intended to target and kill cancer cells while sparing healthy cells. When an antibody attaches itself to the antigen on the surface of the cancer cell, this biochemical reaction triggers a signal in the tumor cell, which internalizes or absorbs the antibody together with the linked cytotoxin into the cell. The cytotoxin is released to kill the cancer cell (Fig. 2). ADCs can also diffuse into adjacent tumor cells even if the cells are target-negative, resulting in cell death termed “bystander killing”. ADCs are currently used for DLBCL targeting a range of antigens and using various payloads (Table 2).

**Table 2:** Summary of major antibody–drug conjugates in R/R DLBCLs and NHLs

Medicine name	Targets	Study	Patients	Treatment	Responses
Lonca	CD19	Phase II LOTIS-2 trial (NCT03589469)	R/R DLBCL (NOS), HGBCL, PMBCL	Monotherapy	ORR/CRR: 48.3% (70/145)/24.1% (35/145) Median DOR/PFS/OS: 10.3/4.9/9.9 mo
		Phase II LOTIS-3 trial (NCT03684694)	Advanced DLBCL	Lonca plus ibrutinib	ORR/CRR in total: 57.1% (20/35)/34.3% (12/35) ORR/CRR in GCB: 76.9% (10/13)/46.2% (6/13) ORR/CRR in non-GCB: 45.5% (10/22)/27.3% (6/22) Median DOR: 5.49 mo (NR in both GCB and non-GCB)
SAR3419	CD19	Phase II (NCT01472887)	R/R DLBCL	Monotherapy	ORR/CRR in total: 43.9% (18/41)/14.6% (6/41) ORR/CRR in refractory to last regimen: 26.7% (4/15)/6.7% (1/15) ORR/CRR in relapsed DLBCL: 53.8% (14/26)/19.2% (5/26) ORR/CRR in primary refractory: 21.4% (3/14)/7.1% (1/14) median DOR/PFS/OS: 4.7/4.4/9.2 mo
BV	CD30	Phase II (NCT01421667)	DLBCL, other B-cell NHL	Monotherapy	ORR/CRR in total DLBCL: 44% (21/44)/17% (8/48) ORR/CRR in refractory DLBCL: 44% (17/39)/15% (6/39) ORR/CRR in relapsed DLBCL: 38% (3/8)/25% (2/8) ORR/CRR in other B-cell NHL: 26% (5/19) 16% (3/19)
		Phase I (NCT02086604)	R/R DLBCL	BV plus Len	ORR/CRR in CD30^**+**^/GCB, 87.5% (7/8)/50% (4/8) ORR/CRR in CD30^**−**^/GCB, 25% (3/12)/17% (2/12) ORR/CRR in CD30^**+**^/non-GCB, 57% (4/7)29% (2/7) ORR/CRR in CD30^**−**^/non-GCB, 70% (7/10)/50% (5/10) median PFS/ OS in total, 10.2/14.3 mo
		Phase III (NCT04404283)	R/R DLBCL	BV plus R2	Clinical trials ongoing
Pina	CD22	Phase II ROMULUS trial (NCT01691898)	R/R DLBCL R/R FL	R-Pina	ORR/CRR in R/R DLBCL, 60% (25/42)/26% (11/42) ORR/CRR in R/R FL, 62% (13/21)/5% (1/21) Median DOR/PFS/OS of R/R DLBCL, 6.2/5.4/16.5 mo Median DOR/PFS/OS of R/R FL, 6.5/12.7/NR mo
Pola	CD79b	Phase II ROMULUS trial (NCT01691898)	R/R DLBCL R/R FL	R-Pola	ORR/CRR in R/R DLBCL, 54% (21/39)/21% (8/39) ORR/CRR in R/R FL, 70% (14/20)/45% (9/20) Median DOR/PFS/OS of R/R DLBCL, 13.4/5.6/20.1 mo Median DOR/PFS/OS of R/R FL, 9.4/15.3/NR mo
		Phase II DCDS4501A trial (NCT02257567)	R/R DLBCL R/R FL	Pola-BR vs. BR	Best responses (INV) ORR, 70% (28/40) vs. 32.5% (13/40) CRR, 57.5% (23/40) vs. 20% (8/40) Median DOR/PFS/OS: 10.3 VS. 4.1 mo/7.6 vs. 2.0 mo/12.4 vs. 4.7 mo
		Phase Ib/II (NCT02600897)	R/R DLBCL	Pola-R-Len	Best responses (INV) ORR/CRR, 74% (36/49)/35% (17/49) Median DOR/PFS/OS, 8.1/6.3/10.9 mo
		Phase Ib/II (NCT02611323)	R/R DLBCL	Pola-Ven-R	Best responses (INV) ORR/CRR, 65% (31/48)/38% (18/48) Median DOR/PFS/OS, 5.8/4.4/11.1 mon
		Phase III POLARGO trial (NCT04182204)	R/R DLBCL	Pola-R-GemOx vs. R-GemOx	Clinical trials ongoing

*R/R* relapsed/refractory, *DLBCL* diffuse large B-cell lymphoma, *NHL* non-Hodgkin lymphoma, *Lonca* loncastuximab tesirine, *SAR3419* coltuximab ravtansine, *BV* brentuximab vedotin, *Pina* pinatuzumab vedotin, *Pola* polatuzumab vedotin, *NOS* not otherwise specified, *HGBCL* high-grade B-cell lymphoma, *PMBCL* primary mediastinal large B-cell lymphoma, *FL* follicular lymphoma, *Len* lenalidomide, *R2* rituximab + lenalidomide, *Ven* venetoclax, *GemOx* rituximab, gemcitabine, and oxaliplatin, *GCB* germinal center B-cell, *ORR* overall response rate, *CRR* complete response rate, *mDOR* median duration of response, *mOS* median overall survival, *mPFS* median progression-free survival, *NR* not reached, *INV* investigator, *mo* months

#### Loncastuximab tesirine

Loncastuximab tesirine (Lonca, ADCT-402) is an ADC comprising a humanized anti-CD19 mAb conjugated to a pyrrolobenzodiazepine dimer cytotoxic alkylating agent tesirine (SG3199) (Fig. 2). Based on the results of a multicenter phase II LOTIS-2 trial, it obtained accelerated FDA approval for R/R DLBCL after two or more lines of therapy [18, 19]. In this study, 145 (79%) heavily treated DLBCL patients were enrolled and received at least one dose of Lonca, including patients with high-risk characteristics for poor prognosis, such as double-or triple-hit lymphoma (DHL or THL), transformed, or primary refractory DLBCL. The ORR was 48·3% (70/145), with 35% of patients achieving CR. The median DOR was 10.3 months, with 13.4 months and 5.7 months for patients with CR and PR, respectively. The median PFS, OS, and relapse-free survival (RFS) were 4.9 months, 9.9 months, and 13.4 months, respectively [19]. A similar ORR was reached for CAR-T therapy patients (46% vs. 48.3%). In addition, the ORR in patients with DHL or THL was 33% (all CR). Besides, the ORR in patients who underwent CAR-T therapy after Lonca was 47% (7/15) with 40% (6/15) of CR [19]. The most common grade 3 or higher TEAEs were neutropenia (26%), thrombocytopenia (18%), and increased gamma-glutamyltransferase (17%). Although TEAEs with a fatal outcome occurred in 6% (8/145) of patients, none were considered related to Lonca. Serious AEs (SAEs) were reported in 39% (57/145) of patients. TEAEs leading to dose modifications or treatment discontinuation occurred in 62% (90/145) of patients. Dose delays were mostly less than 1 week, enabling patients to continue treatment. The interim results of a phase II study of Lonca plus ibrutinib in patients with advanced DLBCL (LOTIS-3) showed encouraging antitumor activity and a manageable safety profile with an ORR 57.1% (34.3% of CR) [20]. Lonca is being evaluated in combination with other active agents, such as ibrutinib (NCT03684694), venetoclax (NCT05053659), and in combination with rituximab versus R-GemOx in a phase III trial in patients with R/R DLBCL (NCT04384484).

#### Coltuximab ravtansine

Coltuximab ravtansine (SAR3419) is another ADC with an anti-CD19 mAb conjugated to a potent cytotoxic maytansinoid, DM4 (tubulin toxin derived from maytansine), via an optimized, hindered, disulfide bond (Fig. 2). In a phase II multicenter study (NCT01472887), the efficacy and safety of SAR3419 were analyzed in patients with CD19^+^ R/R DLBCL. The ORR, CR, and PR rates were 43.9% (18/41), 14.6% (6/41), and 29.3% (12/41), respectively. The median DOR, PFS, and OS were 4.7 months, 4.4 months, and 9.2 months, respectively. The most common grade 3–4 hematologic laboratory abnormalities were neutropenia (25%), lymphopenia (21%), and leukopenia (15%) [21].

#### Brentuximab vedotin

Brentuximab vedotin (BV) is a compound of CD30 mAb linked to cytotoxic moiety monomethyl auristatin (MMAE) and directed against CD30, which disrupts the microtubules resulting in apoptosis of targeting tumor cells (Fig. 2). BV showed promising efficacy in classic Hodgkin’s lymphoma (cHL) and systemic anaplastic large-cell lymphoma (sALCL). The utilization of BV in R/R DLBCL is limited, mainly focused on cases with CD30 expression [22]. In a phase II trial, 49 patients with R/R DLBCL were treated with BV and demonstrated an ORR of 44% with 17% CR [23]. The most common TEAEs were mainly fatigue (55%), diarrhea (43%) and neutropenia (41%). Pyrexia (10%) and pneumonia (9%) were the most frequently occurring SAEs. In addition, a phase I study evaluated the efficacy and safety of the combination of BV with lenalidomide in R/R DLBCL. The ORR was 57% (73% in CD30^+^ DLBCL) with 35% of CR. The median DOR, median PFS, and median OS were 13.1 months, 10.2 months, and 14.3 months, respectively [24]. Combining brentuximab vedotin and rituximab achieved an ORR of 46% with a median follow-up of 2.8 months. TEAEs were similar to those reported in the monotherapy cohort. A phase III study applying BV plus lenalidomide and rituximab to R/R DLBCL after two lines of systemic therapy who were ineligible for hematopoietic stem cell transplantation or CAR-T therapy (ECHELON-3, NCT04404283) is ongoing.

#### Pola

Pola is a second-generation ADC composed of a humanized mAb targeting CD79b conjugated to MMAE through engineered cysteines by a protease-cleavable peptide linker delivering the drug directly into malignant B-cells (Fig. 2) [25]. CD79 is an ideal therapeutic target for antibodies as it is widely and exclusively expressed in most B-cell NHLs, including DLBCLs [26, 27]. In R/R DLBCL, combining Pola with rituximab in a phase II trial resulted in an ORR of 54% with 21% of CR [28]. The median DOR, PFS, and OS were 13.4, 5.6, and 20.1 months, respectively [28]. In R/R DLBCL patients of auto-SCT ineligible, although the Pola-BR group had higher rates of grade 3–4 neutropenia, anemia, and thrombocytopenia (but similar rates of grade 3–4 infections), Pola combined with BR (Pola-BR) resulted in a significantly improved CR rate (40.0% v 17.5%), PFS (median, 9.5 v 3.7 months, HR, 0.36) and OS (median, 12.4 v 4.7 months, HR, 0.42) compared with BR alone [29]. The most common grade 3–4 TEAEs were anemia, neutropenia, and thrombocytopenia in Pola-BR. Peripheral neuropathy (43.6%) was the only reason for Pola dose reduction. Although Pola is an ADC target CD79b, biomarkers analysis showed no relationship between levels of CD79b expression and clinical outcomes. The same results were also observed in different cells of origin and the status of double expressor lymphoma (DEL) [29]. At the 2021 ASCO annual meeting, Diefenbach et al*.* reported the results of a Phase Ib/II study that evaluated the efficacy of Pola-R-Len (Pola + rituximab lenalidomide) in transplant-ineligible R/R DLBCL (GO29834; NCT02600897). With a median follow-up of 9.7 months, the best ORR and CR rate were 74% and 35%, respectively, and the median PFS and OS were 6.3 and 10.9 months, respectively [30]. In addition, the efficacy data from the phase Ib/II trial combining Pola with rituximab and venetoclax showed an investigator-assessed CR rate of 31% and the best ORR of 65%, with median PFS and OS of 4.4 months and 11 months, respectively [31]. A study to evaluate the safety and efficacy of Pola in combination with R-GemOx (rituximab, gemcitabine, and oxaliplatin) compared to R-GemOx alone in R/R DLBCL patients (POLARGO, NCT04182204) is ongoing. In patients with relapsed disease who need a bridge to either CAR-T or auto-SCT, Pola has proven to be a promising agent used in this setting [32, 33]. A phase III trial evaluated the efficacy of a modified regimen of R-CHOP (Pola-R-CHP), compared to standard R-CHOP, in previously untreated intermediate-risk or high-risk DLBCL patients. After a median follow-up of 28.2 months, the Pola-R-CHP group showed improved PFS but not OS, compared to R-CHOP group [34]. On April 19, 2023, the FDA approved R-CHP for adult patients who have previously untreated DLBCL, NOS, or high-grade B-cell lymphoma and who have an International Prognostic Index score of 2 or greater. Based on this, Pola-R-CHP is become the first line of recommendation in NCCN guideline of DLBCL.

Other ADCs, such as anti-CD19 [35], anti-CD20 [36], anti-CD22 [28], anti‑CD25, anti‑CD37, and anti‑CD70, had been investigated previously. Among these, MT-3724, capable of binding to and internalizing against CD20, is a novel engineered toxin body [36]. In a phase Ia/b trial, MT-3724 showed an ORR of 41.7% in R/R DLBCLs with serum rituximab negative [36]. However, others were limited to further use because of the high rate of adverse events [28, 37, 38].

#### BsAbs

**Fig. 3:**
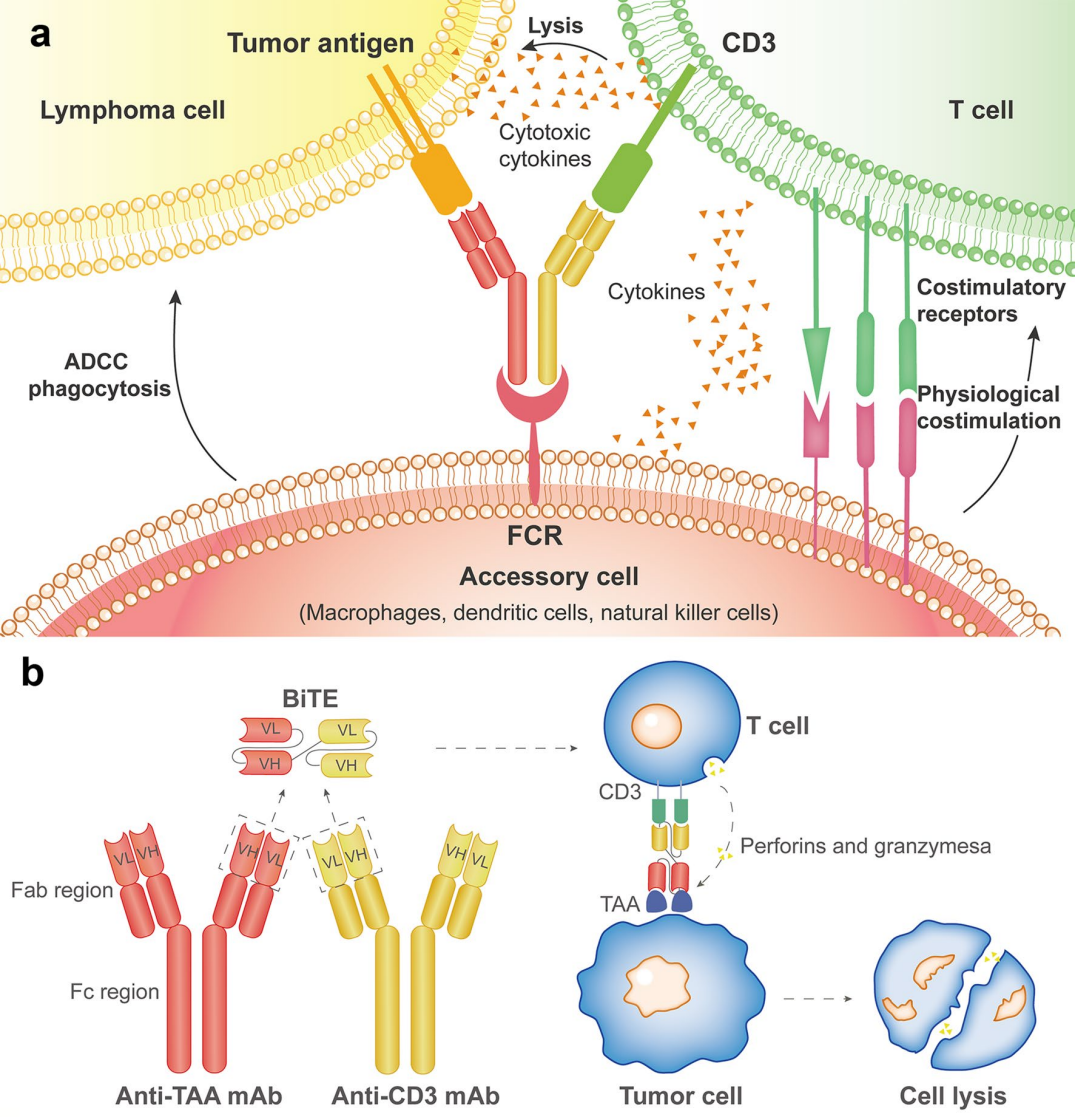
How do bispecific antibodies work. Bispecific antibodies (BsAbs) are engineered to simultaneously bind a cytotoxic cell and a target (a lymphoma cell) to be destroyed. The Fc region binds to cells expressing Fc receptors, like a macrophage, natural killer, or dendritic cell. BsAbs are artificial proteins composed of fragments of two monoclonal antibodies and can bind to two types of antigens (**a**). BsAbs function by bringing targeted tumor cells close to T-cells to allow killing via perforin and granzyme release (**b**). *ADCC* antibody dependent cell-mediated cytotoxicity, *FCR* Fc receptor, *VH* heavy chain variable region, *VL* light chain variable region, *TAA* tumor associated antigen

BsAbs are a new class of immunotherapy agents with the combination of two molecules that recognize two specific epitopes or antigens, both on the tumor and immune cells (such as T-cells, NK-cells, and macrophages). BsAbs also increase cytokine secretion, leading to tumor microenvironment changes (Fig. 3). Recently, different BsAbs have been investigated in R/R DLBCL patients, including those who underwent CAR-T cell treatment, showing promising efficacy and manageable safety profiles with low cytokine release syndrome (CRS) rates and neurotoxicity events [39–42]. Most of the BsAbs under development treating R/R indolent and aggressive B-cell lymphomas engage the CD3 invariant subunit of the T-cell receptor complex, and CD20 (CD20 × CD3 BsAb) or CD19 (CD19 × CD3 BsAb) on lymphoma cells (Table 3).

**Table 3:** Summary of major bispecific antibodies in R/R DLBCLs and NHLs

Medicine name	Targets	Mode of administration	Study	Patients	Treatment	Responses
Blinatumomab	CD19/CD3ε, IgG1	IV	NCT00274742 Phase I	R/R DLBCL	Monotherapy (60 μg/m^2^/day)	ORR/CRR, 54.6% (6/11)/36.4% (4/11)
			NCT01741792 Phase II	R/R DLBCL	Monotherapy (evaluated stepwise or flat dosing)	ORR/CRR, 42.9% (9/21)/19% (4/21)
			NCT02910063 Phase II/III	R/R DLBCL	Monotherapy (stepwise)	ORR/CRR, 37% (15/41)/ 22% (9/41)
Mosunetuzumab	CD20/CD3δε, IgG1	IV or SC	NCT02500407 Phase I/Ib	R/R DLBCL	Monotherapy (dose escalation and standard 3 + 3 escalation)	ORR/CRR, 33% (13/39)/ 21% (8/39)
			NCT02500407 Phase I	R/R B-NHL	Monotherapy (dose escalation)	Aggressive NHL, ORR/CRR, 34.9% (45/129)/19.4% (25/129) Post CAR-T, ORR/CRR, 36.8% (7/19)/26.3% (5/19)
			NCT03677141Phase Ib/II	R/R NHL	M-CHOP (step-up dosing)	ORR/CRR, 86% (6/7)/71% (5/7)
Epcoritamab	CD20/CD3ε, IgG1	SC	NCT03625037 Phase I/II	R/R DLBCL	Monotherapy	12-60 mg, ORR/CRR, 68% (15/22)/45% (10/22) 48 mg, ORR/CRR, 88% (7/8)/38% (3/7) 60 mg, ORR/CRR, 100% (3/3)/100% (3/3)
			NCT03625037 EPCORE NHL-1	R/R DLBCL	Monotherapy (QW, cycle 1–3; Q2W, cycle 4–9; Q4W, cycle ≥ 10)	ORR/CRR, 63% (99/157)/39% (61/157) CAR-T naïve, ORR/CRR, 69% (66/96)/42% (40/96) Post CAR-T, ORR/CRR, 54% (33/61)/34% (21/61)
Glofitamab	CD20/CD3ε (2:1), IgG1	IV	NCT03075696 Phase I	R/R DLBCL	Monotherapy (dose escalation, and dose expansion), Gpt pretreatment	ORR/CRR, 41.4% (30/73)/28.8% (21/73)
			NCT03533283 Phase I/II trial	R/R DLBCL	Glofitamab + Pola (step-up dosing for Glofitamab), Gpt pretreatment	ORR/CRR, 73% (24/33)/51.5% (17/33)
			NCT03075696 Phase II	R/R DLBCL	Monotherapy (step-up dosing), Gpt pretreatment	ORR/CRR, 51.6 (80/155)/39.4% (61/155)
			NCT03467373 Phase Ib	R/R NHL	Glofitamab + R-CHOP, (step-up dosing began in cycle 2 for Glofitamab), Gpt pretreatment	ORR/CRR, 90% (28/31)/77% (24/31)
Odronextamab	CD20/CD3δε, IgG4	IV	NCT02290951 Phase I	R/R DLBCL	Monotherapy (step-up dosing)	CAR-T naïve (All dose), ORR/CRR, 42.6% (23/53)/29.6% (16/54) Post CAR-T (All dose), ORR/CR, 31.1% (14/45)/22.2% (10/45)
Plamotamab	CD20/CD3δε, IgG1		NCT02924402 Phase I	R/R DLBCL	Monotherapy (dose-escalation)	All patients, ORR/CRR, 47.4% (9/19)/26.3% (5/19) Post CAR-T, ORR/CRR, 46.2% (6/13)/30.8% (4/13)

*R/R* relapsed/refractory, *DLBCL* diffuse large B-cell lymphoma, *NHL* non-Hodgkin lymphoma, *B-NHL* B-cell non-Hodgkin lymphoma, *ORR* overall response rate, *CRR* complete response rate, *IV* intravenous, *SC* subcutaneous; *CAR-T* chimeric antigen receptor T cell therapy, *Gpt* obinutuzumab; *Pola* polatuzumab vedotin

### CD19 × CD3 BsAb

#### Blinatumomab

Blinatumomab is the first FDA-approved BsAb for clinical use as a second-line treatment of B-cell acute lymphoblastic leukemia. It is a dual specificity antibody binding to CD19 on target B cells and the CD3e subunit of the T cell receptor. Early clinical trials showed promising results with blinatumomab with R/R B-cell NHL patients. A total of 76 heavily pretreated R/R NHL patients were enrolled in a phase I trial. The ORR in the DLBCL group was 54.6% (CR/CRu 36.4% and PR 18.2%) [43]. In a phase II study, Blinatumomab was tested with 21 heavily treated R/R DLBCL patients. After one cycle of treatment with blinatumomab, the ORR was 43%, including 19% of CR [44]. In a phase II/III study, blinatumomab was administrated to 41 R/R DLBCL patients (including 9 DHL or THL and 15 DELs) who did not achieve CR after salvage chemotherapy, and the ORR and CR rate were 37% and 22%, respectively [45]. Eight (20%) patients (CR or PR) subsequently received SCT (seven for auto-SCT, one for allo-SCT), and 80% of them were alive at nine months. The results suggested blinatumomab was a promising bridge treatment for R/R DLBCL patients who were potentially available for auto/allo-SCT, especially those who failed to respond well to first salvage therapies. The most frequent of TEAEs were neutropenia (10%), anemia (7%), confusional state (7%), aphasia (5%), lower respiratory tract infection (5%), lymphocyte count decreased (5%), neurotoxicity (5%), extremity pain (5%), sepsis (5%), and leukopenia (5%). Grade 3 NE were reported in 24% of patients; all resolved with dexamethasone and/or blinatumomab interruption or discontinuation [45]. Investigations are ongoing, including evaluating blinatumomab in combination with pembrolizumab in R/R DLBCLs (NCT03340766) or as consolidation treatment post-auto-SCT in DLBCLs (NCT03072771) and combination with lenalidomide in R/R NHLs (NCT02568553). Although studies showed blinatumomab was effective in treating R/R DLBCLs, it is limited by significant neurotoxicity and continuous intravenous infusion due to the short half-life.

#### CD20 × CD3 BsAb

CD20 is a validated target in DLBCLs, as shown by the improved PFS and OS of patients treated with R-CHOP compared to CHOP alone [46, 47]. Several BsAbs targeting CD20 × CD3 are in clinical development based on full-length IgG molecules allowing for intermittent dosing [48]. Moreover, they have the advantages of off-the-shelf availability and a prolonged half-life, enabling more convenient usage.

#### Mosunetuzumab

Mosunetuzumab is a fully humanized IgG1 and the first-in-human CD20 and CD3 BsAb developed with intravenous (IV) and subcutaneous (SC) formulations. Single-agent mosunetuzumab was administered IV in 3-week cycles. In a phase I/Ib trial, R/R DLBCL patients treated with mosunetuzumab had an ORR of 33% with 21% CR [49]. At a median follow-up of more than 12 months, all patients with CR remained in remission [49]. The majority of TEAEs occurred during the first cycle. Cytokine release syndrome (CRS) was the most frequently reported drug-related AE, occurring in 21% of patients and mostly occurring with the first dose. All cases of CRS were grade 1–2. Grade ≥ 3 AEs occurred in 52% of patients, of which 22% were considered treatment-related. Only one treatment-related grade ≥ 3 neurotoxicity was reported (grade 3 hepatic encephalopathy). In another phase I dose-escalation study, the ORR was 34.9% with 19.4% CR. For aggressive B-NHL patients, the median DOR was 22.8 months [50]. In patients who were refractory to prior CAR-T therapy (15 with aggressive NHLs and 4 with indolent NHLs), the ORR was 36.8% with 26.3% of CR (two-thirds DLBCLs maintained response by the clinical cutoff date) [50]. At the 2020 ASH annual meeting, Phillips et al*.* reported the safety and efficacy of mosunetuzumab plus CHOP (M-CHOP) in R/R NHLs and newly diagnosed DLBCLs. In the R/R NHLs group, the ORR was 86%, with 71% of CR. In previously untreated DLBCL, the ORR was 96% with 85% of CR [51]. These promising results have led to different new studies [52]. Other clinical trials in R/R DLBCL patients in combination with various drugs, such as atezolizumab (NCT02500407), polatuzumab (NCT03671018), or GemOx (NCT04313608), are ongoing. Some trials are designed in the upfront setting combined with chemotherapy (NCT03677141).

#### Epcoritamab

Epcoritamab (GEN3013) represents the first SC IgG1-based CD20 × CD3 BsAb which binds CD20 antigen on a different epitope in respect of the most common anti-CD20 mAbs. In a phase I/II study, the safety and efficacy of GEN3013 were evaluated. The ORR in R/R DLBCL was 68%, with 45% CR at 12–60 mg doses. At 48 mg, the ORR was 88%, with 38% of CR [53]. No dose-limiting toxic effects or reduction occurred, and the maximum tolerated dose was not reached. The most common TEAEs were pyrexia (69%, with 91% being grade 1–2), primarily associated with CRS (59%, all grade 1–2), and injection site reactions (47%). NEs occurred in 6% (3% each in grade 1 and grade 3) of patients. Recent data from the phase II expansion cohort of the pivotal trial (EPCORE NHL 1) reported at the EHA2022 confirmed the activity of GEN3013, with 38.9% (61/157) receiving prior CAR-T therapy, and 19.7% (31/157) progressed from auto-SCT. About 61% and 83% of patients were primary refractory disease and refractory to the last therapy, respectively. With a median follow-up of 10.7 months, the ORR assessed by PET-CT was 63%, with 39% CR. The ORR and CR rates were 69% and 42% for CAR-T naive patients, whereas 54% and 34% for patients who underwent prior CAR-T therapy [54]. On May 19, 2023, the FDA granted accelerated approval to epcoritamab for R/R DLBCL after two or more lines of systemic therapy. In the front-line setting with high-risk DLBCL, GEN3013 was investigated in combination with R-CHOP. A phase I/II trial reported an ORR of 100% (9/9) without significant CRS [55]. In patients with R/R DLBCL, the phase III EPCORE DLBCL-1 trial investigating the efficacy of the single-agent GEN3013 vs. investigator’s choice chemotherapy (NCT04628494) is ongoing.

#### Glofitamab

Glofitamab is an IgG1-based BsAb antibody with a 2:1 configuration, allowing the bivalent binding to CD20 on B-cells and monovalent binding to CD3 on T-cells [42]. A single dose of GA101 (1000 mg) is preceded to reduce the mature circulating B-cells and minimize the systemic CRS. Glofitamab has been assessed in a phase I trial of heavily pretreated R/R NHL patients (N = 171), including 73 DLBCL patients. The ORR of R/R DLBCLs was 41.1%, with 28.8% of CR. At doses ≥ 10 mg, the ORR and CR rate were 55.3% and 42.1%, respectively. The TEAEs of CRS occurred in 50.3% of patients (grade 3 or 4: 3.5%); 1.2% experienced grade 3 immune effector cell-associated neurotoxicity syndromes (ICAN) [42]. In a phase I/II trial, glofitamab was also investigated with Pola in R/R DLBCL patients. No new safety signals were observed. After a median follow-up of 3 months, the ORR was 73%, with 51.5% of CR [56]. The pivotal phase II expansion trial enrolled R/R DLBCL patients treated with more than two prior therapies. Glofitamab showed an ORR of 51.6%, with 39.4% of CR. At the data cut, the 12-month OS rate was 48%, and 92% of CR patients were alive [57]. On June 15, 2023, the FDA granted accelerated approval to glofitamab for R/R DLBCL after two or more lines of systemic therapy. In the front-line setting, the phase Ib, NP40126 study (NCT03467373) investigated the combination of glofitamab with R-CHOP in R/R NHLs and DLBCLs. The combination therapy was tolerable and safe, with a low CRS rate and no neurotoxicity. All patients (4/4) achieved CR at 30 mg [58]. Encouraging activity and impressive efficacy were observed in heavily pretreated patients, including patients of post-CAR-T therapy [57]. Glofitamab is also being investigated in numerous combination trials for R/R and untreated B-cell NHLs (NCT04408638, NCT04914741, NCT03533283).

#### Odronextamab

Odronextamab (REGN1979) is a fully human IgG4 CD20 × CD3 BsAb with a modified Fc domain being studied in lymphoma. In a phase I study, 71 patients with R/R DLBCL have treated with odronextamab at doses ≥ 80 mg. The ORR was 60% (all CR), and the median DOR was 10.3 months. In patients those refractory to the prior CAR-T therapy, the ORR was 33.3% with 23.8% CR, and the median DOR was 2.8 months. Overall toxicity included pyrexia, CRS, and chills, most commonly, with over 7% of patients experiencing grade 3 or higher CRS and 2.3% of patients suffering from neurologic toxicity [39]. These results led to the ongoing pivotal phase II study for different disease groups (NCT03888105) and the combination with cepilimab (NCT02651662).

#### Plamotamab

Plamotamab (XmAb13676) is another humanized CD20 × CD3 BsAb modified for better potency and safety. Update results of an ongoing dose-escalation study (NCT02924402) in R/R DLBCLs were reported at the 2022 ASH annual meeting. The ORR was 47.4%, with 26.3% of them achieving CR. For patients posted CAR-T therapy, the ORR was 46.2%, and the CR rate was 30.8% [59]. Among these, 62.5% of cases experienced CRS, with 5.0% experiencing grade ≥ 3 CRS. No related neurotoxicity > Grade 2 has been observed.

### CAR-T cell Therapy

**Fig. 4:**
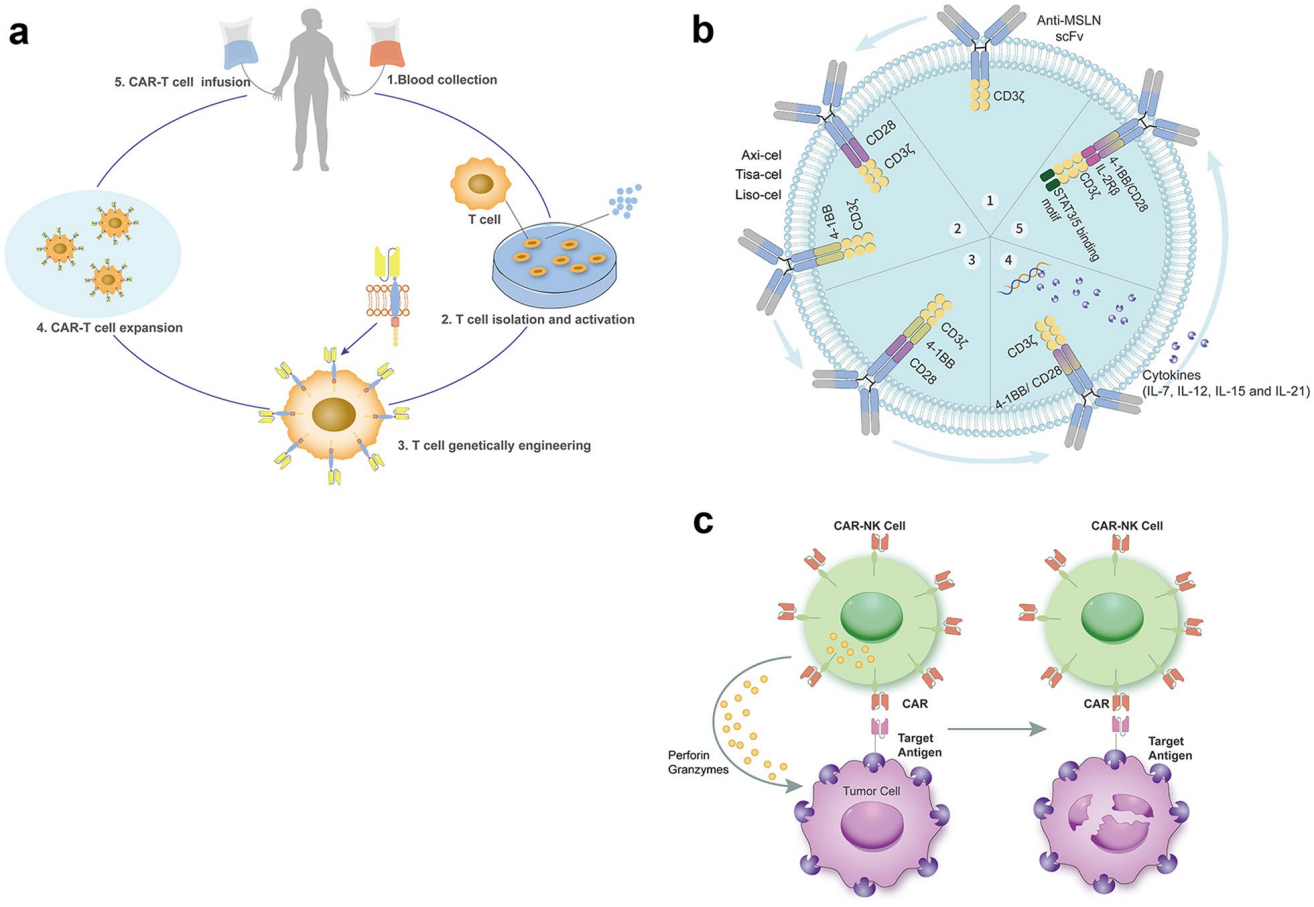
The usage and progress of CAR-T cell therapy. In CAR T-cell therapy, the patient's T cells are collected and sent to a lab. In the lab, they are genetically modified to recognize target lymphoma cells. These genetically modified T cells are named CAR-T cells. After that, the CAR-T cells are expanded in the lab until there are enough to treat the lymphoma cells. Then, CAR-T cells are returned to the patient, like a blood transfusion. When they recognize the lymphoma cells in the body, the CAR-T cells are activated and kill the lymphoma cells (**a**). There are currently five generations of CAR-T cell products. The first-generation, composed of scFv and CD3ξ, is a single chain approach based on the scFv, which joins the antibody's heavy and light variable gene segments with a flexible linker. Second-generation CARs contain the scFv and CD3ξ components present in the first-generation together with a costimulatory domain, which markedly increases T-cell proliferation and interleukin -2 secretion. Axi-cel contains a CD28 costimulatory domain, while Tisa-cel and Liso-cel contain the 4-1BB costimulatory domain. The third-generation CARs contain both CD28 and 4-1BB and have superior expansion and longer persistence than the second-generation CARs. Fourth-generation CARs incorporate a transgenic cytokine sequence and counteract the immunosuppressive microenvironment in tumors. The fifth-generation CARs encode a truncated cytoplasmic domain of IL-2Rb and a STAT3- binding YXXQ motif together with scFv targeting CD19, CD3z, and CD28 domains, which show better proliferation and cytokine polyfunctionality compared to second-generation CARs (**b**). NK cells do not require HLA matching like T cells. It makes “off-the-shelf” NK cell therapy a viable option. CAR NK cells will release perforin and granzymes to kill tumor cells (**c**). Most CAR-T therapies consist of autologous T cells, whereas CAR-NK cell therapies can be generated from allogeneic donors

CAR-T cell therapy is one of the most effective treatments for B-cell malignancies, including DLBCLs. The universal presence of CD19, CD20, and CD22 antigens on malignant B-cells makes them the perfect targets for cellular therapies (Fig. 4a). Anti-CD19 CAR-T cell therapy is constitutive of autologous T lymphocytes redirected against CD19 antigen on B cells by introducing a CAR with a replication-incompetent retroviral vector (Fig. 4b). This treatment platform comprises lymphodepletion chemotherapy followed by a single CAR-T cell infusion.

### CAR-T cell as later lines (≥ third line) of therapies in R/R DLBCLs

Three CAR-T products are currently available for R/R DLBCL patients. Axicabtagene ciloleucel (Axi-cel), tisagenlecleucel (Tisa-cel), and lisocabtagene maraleucel (Liso-cel), with some structural differences mainly derived to a different costimulatory domain (CD28 for Axi-cel, 4-1BB for Tisa-cel and Liso-cel) and a unique, balanced CD4^+^/CD8^+^ T cells ratio for Liso-cel, showed promising clinical efficacy in R/R DLBCLs after at least two prior lines of therapies. In the three pivotal trials ZUMA-1 (Axi-cel), JULIET (Tisa-cel), and TRANSCEND NHL 001 (Liso-cel), these three CAR-T therapies showed deep and durable responses, with the ORR ranging from 53 to 83% and 39 to 58% of CR. The median PFS was 2.9 to 6.8 months, and the median DOR was 11.1 months to NR among different trials [60–62] (detailed efficacies and TEAEs were shown in Table 4). In fact, according to the SCHOLAR-1 study, patients with R/R DLBCL after second-line therapy were extremely poor. The ORR was only 26% to the later lines of treatment, with a median OS of only 6.3 months. Due to the remarkable efficacy of CAR-T therapy, the FDA and EMA have approved the usage of Axi-cel, Tisa-cel, and Liso-cel for adult patients with R/R DLBCL as the third or later lines of therapies. Since there is a lack of an adequate comparison for efficacy and safety among the above three studies, Bachy et al. compared the outcomes in 809 patients with R/R DLBCL who received commercial CAR-T cells therapies for either Axi-cel or Tisa-cel (NCT04328298) [63]. The best ORR and CR rates were 80% and 60% versus 66% and 42% for patients treated with Axi-cel and Tisa-cel [63]. One-year PFS (46.6% vs. 33.2%) and OS (63.5% vs. 48.8%) were significantly improved after Axi-cel infusion cases compared to Tisa-cel treated ones [63]. However, Grade 1/2 (but not grade ≥ 3) CRS was more frequent in Axi-cel compared to Tisa-cel. All grade ICANS were significantly more frequent in Axi-cel than in Tisa-cel [63]. Using matching-adjusted indirect treatment comparison (MAIC), Cartron et al. showed that Liso-cel had statistically significant greater efficacy than Tisa-cel (ORR, 72.7% vs. 51.6%; CRR, 53.1% vs. 39.8%). In the primary analysis (matched and adjusted for six factors) and sensitivity analysis (matched and adjusted for all available clinical factors except for bridging therapy), Liso-cel had an ORR of 74.7% (effective sample size, ESS = 164) and 80.8% (ESS = 37.3), respectively [64]. In another study, Maloney et al. compared the efficacies between Liso-cel and Axi-cel and showed that Liso-cel had greater efficacy and a more favorable safety profile than Axi-cel [65].

**Table 4:** Summary of primary CD19 CAR-T products in R/R DLBCLs

Lines	Study	CAR-T cells	Patients	Outcomes	Toxicities of CAR T
≥ 3	ZUMA-1 NCT02348216	Axi-cel	DLBCL, PMBCL, tFL, HGBCL	ORR/CRR, 83%/58% Median DOR, 11.1 mo Median OS, 25.8 mo Median PFS, 5.9 mo	≥ Grade 3 CRS 11% ≥ Grade 3 NEs 32%
≥ 3	JULIET NCT02445248	Tisa-cel	DLBCL, tFL, HGBCL	ORR/CRR, 53·0%/39% Median DOR, NR Median OS, 11.1 mo Median PFS, 2.9 mo	≥ Grade 3 CRS 11% ≥ Grade 3 NEs 32%
≥ 3	TRANSCEND NHL 001 NCT02631044	Liso-cel	DLBCL, FL3B, PMBCL, tFL; DLBCL transformed from iNHL, HGBCL	ORR/CRR, 73%/53% Median DOR, NR Median OS: 21.1 mo Median PFS: 6.8 mo	≥ Grade 3 CRS 2% ≥ Grade 3 NEs 10%
≥ 2	ZUMA-7 NCT03391466	Axi-cel vs. SOC	DLBCL, tFL, HGBCL with or without MYC and BCL2 and/or BCL6 rearrangement, THRBCL	ORR, 83% vs. 50% CRR, 65% vs. 32% Median EFS, 8.3 vs. 2 mo Median PFS, 14.7 vs. 3.7 mo Median OS, NR vs. 35.1 mo	≥ Grade 3 CRS 6% ≥ Grade 3 NEs 21%
≥ 2	TRANSFORM NCT03575351	Liso-cel vs. SOC	DLBCL, DLBCL transformed from iNHL, FL3B, HGBCL with MYC and BCL2 and/or BCL6 Rearrangement, DHL, THL, PMBCL, THRBCL	ORR, 86% vs. 48% CRR, 61% vs. 36% Median DOR, NR vs. 14.5 mo Median EFS, 10.1 vs. 2.3 mo Median PFS, 14.8 vs. 5.7 mo Median OS, NR vs. 16.4 mo	≥ Grade 3 CRS 1% ≥ Grade 3 NEs 4%
≥ 2	BELINDA NCT03570892	Tisa-cel vs. SOC	DLBCL, HGBCL, FL3B, transformed from previous lymphoma	ORR, 46.3% vs. 42.5% CRR, 28.4 vs. 27.5% Median EFS, 3 mo for both Median OS, 16.9 vs. 15.3 mo	≥ Grade 3 CRS 5.2% ≥ Grade 3 NEs 1.9%
≥ 2	PILOT-017006 NCT03483103	Liso-cel (JCAR017))	R/R DLBCL ineligible of auto-SCT	ORR/CRR, 80%/54% Median DOR, 12.09 mo Median PFS, 9.3 mo Median EFS, 7.23 mo Median OS, NR	≥ Grade 3 CRS 1.6% ≥ Grade 3 NEs 4.9%

*Axi-cel* axicabtagene ciloleucel, *Tisa-cel* tisagenlecleucel, *Liso-cel* lisocabtagene maraleucel, *SOC* standard of care, *CAR-T* chimeric antigen receptor T cell therapy, *R/R* relapsed/refractory, *DLBCL* diffuse large B-cell lymphoma, *PMBCL* primary mediastinal large B-cell lymphoma, *tFL* large-cell transformation from follicular lymphoma, *HGBCL* high-grade B-cell lymphoma, *THRBCL* T-cell– or histiocyte–rich large B-cell lymphoma, *FL3B* follicular lymphoma grade 3B, *iNHL* indolent non-Hodgkin lymphoma, *DHL/THL* double-or triple-hit lymphoma, *R/R* relapsed/refractory, *auto-SCT* autologous hematopoietic stem cell transplant, *ORR* overall response rate, *CRR* complete response rate, *mDOR* median duration of response, *mOS* median overall survival, *mPFS* median progression-free survival, *EFS* median event free survival, *CRS* cytokine release syndrome, *NR* not reached, *NEs* neurotoxicity events, *mo* months

### CAR-T cell as second-line therapies for R/R DLBCLs

The impressive results of CAR-T therapy as the third line prompted clinicians to test them as a second-line treatment for R/R DLBCLs. Three large randomized phase III trials have been conducted comparing the above three CAR-T products with salvage platinum-based chemotherapy regimens followed by auto-SCT (named standard of care, SOC) in patients refractory to front-line treatment or relapsed within 12 months [66–68]. ZUMA-7 (Axi-cel vs. SOC) and TRANSFORM trials (Liso-cel vs. SOC) randomized 359 and 194 patients, respectively, and demonstrated the superiority of the two CAR-T products in respect of SOC, both in terms of treatment responses and survival [66, 67] (Table 4). In contrast, the BELINDA trial showed no survival differences between Tisa-cel and SOC [68] (Table 4). The positive results from ZUMA-7 and TRANSFORM trials established a new therapy breakthrough for R/R DLBCLs. On April 1, 2022, the FDA approved Axi-cel for adult patients with DLBCL who are refractory to first-line immunochemotherapy (FLIC) or relapses within 12 months of FLIC. On June 24, 2022, the FDA approved Liso-cel for patients who refractory to FLIC or relapsed within 12 months; or those who are not eligible for auto-SCT due to comorbidities or age. Westin et al*.* reported the subgroup analysis of patients ≥ 65 years in ZUMA-7. The subgroup analysis ZUMA-7 showed that Axi-cel (compared to SOC) was an effective second-line curative-intent therapy (ORR: 88% vs. 52%; CR rate: 75% vs. 33%) with a manageable safety profile (Grade ≥ 3 AEs: 94% vs. 82%) and improved efficacy for old patients (≥ 65 years) with R/R DLBCL [69].

### Strategies for post-CAR-T therapy with R/R DLBCLs

Despite the high rate of CRs seen with CAR-T therapies, only 30–40% of patients achieve durable remissions [60, 62]. Relapse post-CAR-T therapies showed poor prognoses and were regarded as the development of resistance. The significant patterns of resistance to CAR-T therapies have been investigated recently. The impaired death receptor signaling and dysfunctional CAR-T cells result in the lack of response to CAR-T cells (primary resistance, antigen-positive relapse). Loss of CD19 antigen and low quality of CAR-T cell expansion or T cell exhaustion cause disease progression after the response (secondary resistance, antigen-negative relapse) [70].

T-cell dysfunction with decreased functional T cells is more common in patients who have been heavily treated. Allogeneic CAR-T-cells (off-the-shelf CAR-T) may overcome these obstacles. Multiple novel CAR-T cell therapies are under investigation, including PBCAR0191 and ALLO-501 (allogeneic CD19-directed CAR-T), PBCAR20A, MB-106, C-CAR066, and LUCAR-20S (CD20-directed CAR-T), and CAR22 (autologous CD22-directed CAR-T, sequential CD22/CD19 CAR-T therapy) [6, 71, 72].

Recent studies indicated that persisted immunosuppressive tumor microenvironments (TMEs) are essential during disease progression before and after CAR-T cell infusion. Yan et al*.* performed single-cell RNA sequencing on lymphoma samples collected from patients during treatment and follow-up. They found that the percentages of M2 macrophages were much higher in the patient progressed than in remission (48.2% vs. 29.2%) [70]. They indicated that the M2-subtype macrophages could decrease the cytotoxic activity of CAR-T cells by inhibiting the cytokine production, cytotoxic ability, and proliferation of CAR-T cells [70]. In addition, they also found that M2 macrophages could suppress the anti-tumor functions of effector T cells by metabolic reprogramming in the progressed patient [70]. Thus, targeting TMEs and metabolism might be possible to reverse CAR-T cell therapy's resistance mechanisms.

It is well known that patients with positive PD‐L1/PD‐1 expression have poor prognoses. Both preclinical and clinical studies showed that DLBCL cells with a high PD‐1/PD‐L1 interaction did not benefit from CAR-T cell therapy, which could be reversed by PD-1 blockade [73]. Immune checkpoint upregulation (PD-1/PD-L1), indicating T cell exhaustion, has been observed in patients after CAR T-cell infusion, suggesting that PD-1/PD-L1 inhibition may represent an important therapeutic target in this setting [74]. ZUMA-6 investigated the safety and efficacy of Axi-cel in combination with atezolizumab in refractory DLBCL patients (NCT02926833). CAR-T cell expansion was two-fold higher than observed in the ZUMA-1 trial. At 4.4 months follow-up, the combination had a manageable safety profile with an ORR of 90% (9/10), and 60% of cases achieved CR [75]. Two recent studies also suggested that both nivolumab and pembrolizumab had the potential therapeutic benefit of reversing CAR-T cell exhaustion in R/R DLBCL patients [76, 77]. Mu et al*.* demonstrated the combination and maintenance treatment with a PD-1 inhibitor in PD-1 positive R/R DLBCL patients who achieved CR/PR after CAR-T therapy experienced prolonged survival [78]. Another study indicated immune checkpoint inhibitor (ICI) was an effective salvage strategy for primary mediastinal B-cell lymphoma (PMBL) and those with late relapse after CAR-T therapy, except for early relapse after CAR-T cell therapy [79]. Using engineering strategies by co-expressing a chimeric PD-1/CD28 switch-receptor, Liang et al*.* reported that CD19-PD-1/CD28-CAR-T cells exhibited potential clinical efficacy as a salvage treatment after failure of conventional CD19-directed CAR-T therapy [80]. Although the great potential of ICI as one of the salvage strategies for CAR-T treatment failure, alternative approaches are still needed to improve the outcomes of CAR-T cell treatment. Bruton tyrosine kinase (BTK) inhibitors or BsAbs (mentioned above), in conjunction with CAR-T therapy, might be another choice for patients who have experienced CAR-T therapies [52, 81, 82]. Besides, radiotherapy (RT) combined with CAR-T therapy induced better outcomes in patients with localized relapses, especially those who received salvage RT [83].

Various strategies are being explored to decrease the possibility of resistance through augmenting T cell activity or targeting different lymphoma antigens. T cell receptor (TCR)-engineered T cell therapy is another cellular immunotherapy which induces more durable signal activation with mild treatment-related toxicities [84]. By replacing the antigen recognition domain of TCR, Li et al*.* developed a novel CD19-specific γ/δ TCR-T cells, which could induce rapid responses and durable CR in patients with R/R DLBCL [85]. With a median follow-up of 34 months, the ORR was 87.5% (7/8), with 75% (6/8) achieved CR. The 3-year OS, PFS and DOR were 75.0%, 62.5%, and 71.4%, respectively [85]. Novel dual-antigen targeting by CAR-T cells (e.g. CD19/20 or CD19/CD22 target antigens) is currently being investigated, including combining dual-antigen CAR-T with an ICI [86–88]. In a phase I/II trial, the first bicistronic anti-CD19/CD22 CAR-T cells AUTO3, followed by pembrolizumab, showed acceptable safety profiles in R/R DLBCL patients. The ORR was 75%, with 63% of cases achieving CR [88]. It was reported that decitabine could upregulate tumor-associated antigens and increase the expansion of CAR-T cells [89]. Decitabine containing lymphodepletion might improve the clinical efficacy and prolong PFS in R/R DLBCL patients who received anti-CD19/CD22 CAR-T treatment [90].

Compared to CAR-T cells, CAR-NK cells represent another more appealing alternative strategy with an easy and rapid production process and less toxicity (Fig. 4c). In a CAR-independent manner, CAR-NK cells can kill lymphoma cells by their native receptors and avoid antigen escape [91]. In a phase I/II trial, eleven patients with R/R CD19-positive malignancies (including 2 DLBCL patients) showed an ORR of 73% (8/11), with 63% (7/11) cases achieving CR. According to data from a phase I/II trial, NK cells expressing anti-CD19 CAR and interleukin-15 resulted in responses in 73% (8/11) patients, with 64% of CR [92]. The responses were rapid without developing CRS, neurotoxicity, or graft-versus-host disease. Besides, infused CAR-NK cells expanded and persisted at low levels for at least one year after infusion [92]. In April 2021, the FDA approved the first off-the-shelf CD19-directed CAR-NK cell (NKX019) for treating R/R B-cell malignancies. Other clinical trials assessing the safety and efficacy of CAR-NK cells were under investigation (NCT04245722, NCT05020678, NCT04887012). Thus, the HLA-matched or mismatched NK cells originating from an allogeneic source may enable streamlining of the production process and universal access [92].

The generation of the cellular products requires an adequate absolute lymphocyte count, which could be overcome by moving up the CAR-T therapies to the second line, as tested in ZUMA-7, BELINDA and TRANSFORM trials, or eventually in the first line as designed in ZUMA-12 study, in which Axi-cel have been tested as part of front-line treatment. This phase II trial enrolled high-risk DLBCL patients with positive interim PET results after two cycles of chemo-immunotherapy. The ORR was 89%, with 78% of cases achieving CR. After a median follow-up of 15.9 months, 73% of patients remained in remission. The median DOR, event-free survival (EFS) and PFS were not reached. An estimated 1-year PFS and OS rates were 75% and 91%, respectively [93]. Thus, moving CAR-T therapy to the front setting is becoming a trend in clinical practice.

### Predictive markers for CAR-T cell therapy in R/R DLBCL

Limited durability of response and prevalent toxicities remain the major problems of CAR-T cell therapy. Identifying patients who can benefit from this treatment and who have a high likelihood of recurrence, treatment-related toxicity, and death would help us make treatment decisions. Many factors related to prognosis and efficacy were reported in the literature, including patient features, tumor characteristics and product composition.

In an extensive multicenter retrospective RWE analysis, Shouse et al*.* identified a simplified CIRS-based index predicting outcomes in patients with DLBCL treated by CAR-T cell therapy. The severe comorbidity group (CIRS score ≥ 3, termed Severe4) was independently associated with shorter PFS and OS. Besides, Severe4 was strongly related to relapse-related mortality [94]. These findings suggested that CIRS assessment helped predict treatment efficacy and toxicities of CAR-T cell therapy and should be part of SOC in those patients. Based on the basic ^18^F-FDG PET/CT information before and after CAR-T cell infusions, Winkelmann et al*.* introduced the International Metabolic Prognostic Index, which could predict prolonged PFS with patients of low risk compared to intermediate/high risk [95]. Besides, higher risk (high IPI index), poor performance status, and high levels of lactic dehydrogenase, C-reactive protein, IL-6, and ferritin in peripheral serum blood were considered negative predictive factors of CAR-T therapy. In contrast, increased IL-7, IL-15, and monocyte chemoattractant protein-1 were reported as positive predictive factors [96].

The genetic heterogeneity of R/R DLBCL patients who can benefit from CAR-T cell treatment is still unclear. In a recent study, Shi et al*.* reported the genetic differences in R/R DLBCL who received CAR-T therapy [97]. They indicated that only *TP53* gene alterations were the only factors predictive of inferior CR rate. Though DLBCL patients carrying *TP53* and *DDX3X* mutations had a shorter OS after CAR-T infusion than those with wild type, CAR-T cell treatment improved survival in patients carrying TP53 mutations [97]. Similarly, MCD- and EZB-like subgroups showed a benefit of OS after CAR-T treatment. In addition, CAR-T cell treatment might overcome the adverse prognosis of DHL/THL [97]. For R/R DLBCL patients after CD19/CD22 CAR-T therapy, using whole-exon sequencing, Wang et al*.* found that germline genes variants were significantly enriched in patients who failure to CAR-T therapy, especially with *UNC13D* mutations and *CX3CR1*^I249/M280^ variants, which might be used as factors to predict of T cell dysfunction associated with the primary resistance mechanism [98]. In addition, different ctDNA concentrations and ctDNA mutations pre- and post-CAR-T infusion could help determine prognosis [96].

To evaluate the markers in peripheral blood and clinical information of DLBCL patients who underwent CAR-T cell therapy, Worel et al*.* showed a low frequency of differentiated CD3^+^CD27^−^CD28^−^ T cells at leukapheresis predicted favorable response to CAR-T cell treatment which was independently associated with the ORR [99]. CAR-T cells expressing PD-1, TIM-3 or LAG-3 were supposed to predict treatment failures. In contrast, CAR-T cells enriched in CD8^+^CD27^+^PD-1^−^ T cells, CCR7^+^CD27^+^CD8^+^ T cells, and CD45RA^+^CCR7^+^ T cells were probably achieving clinical responses in candidate patients [96].

### Immune checkpoint inhibitors (ICIs)

**Fig. 5:**
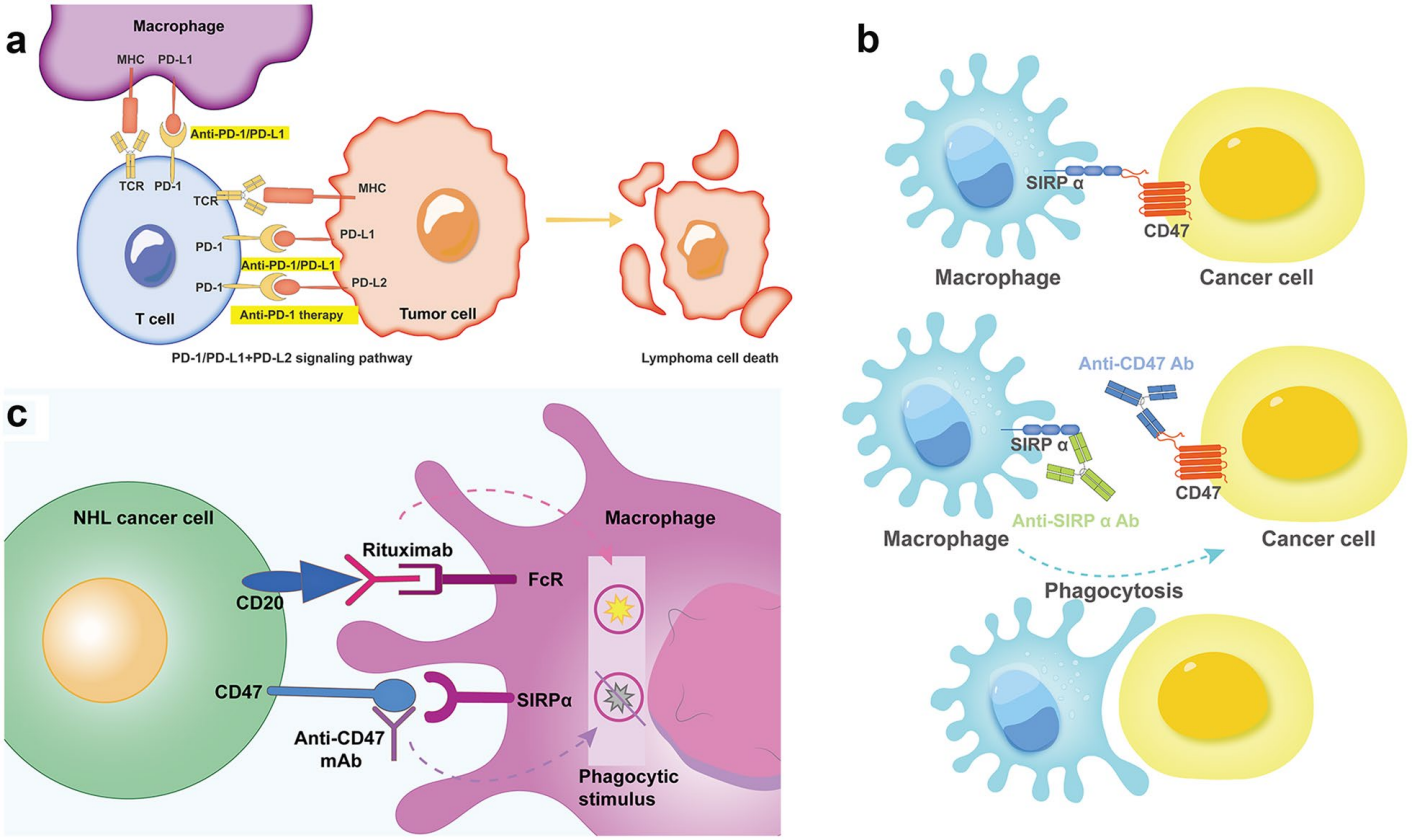
The mechanism and usage of immune checkpoint inhibitors. PD-1/PD-L1 binding inhibits T cell killing of lymphoma cells. Blocking PD-1 and PD-L1 allows T cell killing, APC-T cell interaction, and T cell stimulation in a lymphoma microenvironment (**a**). When SIRPα interacts with its ligand CD47 on tumor cells, SIRPα undergoes tyrosine phosphorylation and recruits the protein tyrosine phosphatases. These phosphatases inhibit the ability of prophagocytic receptors to trigger phagocytosis when ligands are present on tumor cells. Blocking CD47-SIRPα signaling with an anti-CD47 or SIRPα monoclonal antibody enhances macrophage-mediated phagocytosis of lymphoma cells (**b**). Anti-CD47 monoclonal antibody synergises with Rituximab when lymphoma cells double express CD20 and CD47 proteins (**c**)

PD-1 is expressed mainly in the activated T-cells, B-cells, and monocytes [100]. It regulates the T-cell-mediated immune response through binding to its ligands, PD-L1 and PD-L2 (Fig. 5a). ICIs have revolutionized the treatment of solid tumors with PD-L1/2 expression and have become the standard of care for melanoma [101, 102], lung cancer [103, 104], triple-negative breast cancer [105, 106], and urological tumors [107, 108]. In cHLs, recurrent copy gains of gene loci on chromosome 9p24 enhanced activator PD-L1/2 expression [109, 110]. In addition, Epstein Barr virus (EBV) infection and an indication of EBV-derived latent membrane protein one has increased the expression of PD-L1 and PD-L2. It is associated with a shorter PFS [111–114]. However, patients with higher levels of PD-L1 expression driven by genetic alterations in 9p24.1 and intact expression of MHC-II had superior outcomes after PD-1 blockade [115]. Thus, PD-1 blockade by the anti-PD-1 antibodies nivolumab [116–118] and pembrolizumab [119–121] has shown promising results in relapsed and newly diagnosed cHL patients.

Unlike cHLs, those exhibiting T-cell inflamed phenotypes, DLBCLs mostly exhibit T-cell noninflamed phenotypes [109]. DLBCL cells do not frequently express PD-L1 [113]. Despite the impressive results of ICIs in treating solid tumors and cHLs, inhibition of the PD-1/PD-L1 axis has led to less efficacy in R/R DLBCLs [122, 123]. T-cell inflamed lymphomas share many characteristics, including prominent immune cell infiltration, frequent mutations resulting in constitutive NF-κB pathway activation, and EBV infection [109]. In DLBCLs, PD-L1 expression has been identified only in some patients [124]. DLBCL patients with germinal center B-cell (GCB) subtype and high-grade B-cell lymphomas, which lack of above features, may be particularly resistant to ICIs [109]. However, PD-L1 alterations occur more frequently in some DLBCL subtypes, such as PMBLs [125] and EBV-positive DLBCLs [126], which may predict favorable responses to ICIs. The phase 2 KEYNOTE-170 trial showed meaningful responses with durable remissions with pembrolizumab monotherapy in patients with R/R PMBLs. This result led to the FDA approval of pembrolizumab in R/R PMBLs after two or more prior lines of therapy [125]. Similar results were seen in EBV-positive DLBCLs [109, 127]. EBV-positive DLBCL is most common in elderly patients but can occur in younger patients. With age, the balance between the inflammatory and anti-inflammatory deteriorate gradually. This imbalance leads to a chronic pro-inflammatory status, called physiological immunosenescence, which can facilitate lymphomagenesis [128]. In contrast, immune escape is more common in younger EBV-positive patients. In addition, it was reported that CD30 positivity is widespread in EBV-positive DLBCL cases, which is a potential candidate for BV [129]. Thus, combining BV and ICIs might be an attractive or optimized second-line strategy for R/R DLBCL patients with both CD30 and EBV- positive, which has already been assessed in R/R NK/T-cell Lymphoma (NCT05316246).

Whether PD-L1 expressed by DLBCL cells or host cells is predictive of the immunotherapeutic efficacy of ICIs remains unknown. Kiyasu et al*.* evaluated the impact of PD-L1 positivity on tumor cells and their microenvironment (mPD-L1) in DLBCLs. PD-L1 and mPD-L1-positive DLBCLs were significantly associated with the non-GCB subtype and EBV positivity [130]. They also found that PD-1-positive tumor-infiltrating lymphocytes (TILs) were significantly higher in GCB-type tumors and lower in mPD-L1-negative and PD-L1-positive DLBCLs. Patients with PD-L1-positive on DLBCL cells but not mPD-L1-positive had inferior OS than patients with PD-L1-negative on DLBCL cells when treated with standard immunochemotherapy [130]. Our team also recognized the importance of the interaction between tumor cells and their microenvironment. When the function of the microenvironment was impaired, DLBCL patients with PD-1-positive on CD8-positive T cells or PD-L1-positive on T cells and macrophages had significantly poorer survival. In contrast, DLBCL patients with PD-L2 positivity and patients with PD-L1 positivity on CD20-positive cells proximal to PD-1-positive CD8-positive T cells with low PD-1-positive percentage of CD8-positive T cells were associated with superior survival [131]. In addition, tissue PD-L1-positive and T-cell-derived PD-1-positive have significant adverse impacts only in patients with high T-cell infiltration, which suggests the benefit of PD-1/PD-L1 blockade therapies only in patients with sufficient T-cell infiltration [132]. However, the correlation between ICIs and PD-L1 expression was still controversial [133]. ICIs monotherapy has disappointed R/R DLBCLs in the last several years [134]. In a phase 1 study, the efficacy of nivolumab was evaluated in eleven R/R DLBCL patients. The ORR was 36%, with 18% CR [135]. In a large phase 2 study, R/R DLBCLs ineligible for auto-SCT (N = 34) or relapse from auto-SCT (N = 87) received nivolumab 3 mg/kg every 2 weeks. At a median follow-up of 9 months, the ORR was only 3% (all PR) and 10% (3% of CR), respectively [122]. Thus, PD-1/PD-L1 inhibitors are mainly studied with other therapeutics in R/R DLBCL patients. In a phase 1b/2 study, Herrera et al. investigated the combination of ibrutinib + durvalumab in R/R DLBCL patients (GCB DLBCL N = 16, non-GCB DLBCL N = 16, unspecified DLBCL N = 2). In the R/R DLBCL subgroup, however, the combination led to an ORR of 13% in the GCB subtype and 38% in the non-GCB subtype [136]. Several early clinical trials have shown a modest activity of atezolizumab in combination with various therapeutic agents in R/R DLBCL patients [137, 138]. At the front-line setting, pembrolizumab was tested with R-CHOP in 30 patients with DLBCL and resulted in an ORR of 90% (77% of CR), with a 2-year PFS of 83% at a median follow-up of 25.5 months. Longer PFS was seen in patients with higher PD-L1 expression [139]. Atezolizumab, in combination with six cycles of R-CHOP followed by 12 months of consolidation, was evaluated in 42 untreated advanced DLBCL patients. The CR rate was 77.5% at the end of induction, and the three-year PFS and OS were 77.4% and 87.2%, respectively [140]. Early results from clinical trials combining PD-1/PD-L1 inhibitors, atezolizumab, avelumab, and durvalumab, with chemo-immunotherapy have been reported [141–143]. When CAR-T cells were exposed to the antigen in vivo, a significant up-regulation of PD-1, LAG-3, and TIM-3 was found, which indicated CAR-T cell exhaustion (mentioned above). Interestingly, when co-culturing CAR-T cells with pembrolizumab, the viability of CAR-T cells was restored, suggesting a protective effect of ICIs on CAR-T cell functions [144]. Several trials evaluating CAR-T cells' combination with ICIs in R/R DLBCL are ongoing [77, 145].

Although PD1/PD-L1 blockade still seems unsatisfactory in R/R DLBCLs, CD47, considered a macrophage checkpoint, might change the landscape in R/R DLBCLs. CD47 is extensively overexpressed in cancers and prevents tumor cells from phagocytosis and promotes tumor progression by activating the SIRPα-CD47 axis to avoid immune surveillance [146] (Fig. 5b). CD47 expression level is independently correlated with poor clinical outcomes in patients with hematological malignancies [147]. CD47 upregulation on malignant cells reveals immune evasion and drug resistance, which were detected in 53.7% of patients with DLBCL [147]. The first-in-class CD47-directed mAb, magrolimab (Hu5F9-G4), has demonstrated efficacy in patients with NHL in early-phase clinical investigation. Moreover, Hu5F9-G4 was shown to synergistically augment the activity of rituximab and affect lymphoma in preclinical models (Fig. 5c). In a phase 1b study, heavily pretreated patients with R/R DLBCL receiving the combination of Hu5F9-G4 and rituximab (NCT02953509) experienced durable disease control (ORR: 40%; CR: 33%) and rare dose-limiting side effects. However, the best ORR was seen in patients with activated B-cell (ABC)-DLBCL than GCB-DLBCL (67% vs. 17%) [148]. Chauchet et al*.* reported the efficacy of NI-1701 (one of the novel BsAbs targeting CD47 × CD19) in a mouse model, which indicated that NI-1701 could transform the TME to an anti-tumorigenic state and enhance dendritic cell-mediated phagocytosis [149]. NI-1701 is currently being evaluated alone or in combination with ublituximab in patients with R/R B-cell Lymphomas (NCT04806035). Moreover, studies and bioinformatics analyses indicated that CD47 is associated with other DLBCL-related genes, such as PD-L1, LAG-3, TIM-3, and CD4 [146]. Dual blockade of CD47 and PD-L1 may be another potential synergistic therapy that can elicit both innate and adaptive immune responses against tumors [150], which is worth investigating in clinical trials (NCT04328831). LAG-3 and TIM-3 are the most frequently reported genes in DLBCLs, and they are closely related to CD47 as immune checkpoints [146]. Several attempts have been made to target other checkpoint inhibitors such as LAG-3, TIM-3, TIGIT, and VISTA [151]. Other CD47 mAbs showed activity in preclinical models and are being studied in phase 1 studies [152]. TTI-621, a CD47 decoy receptor that targets CD47/SIRPα, is being evaluated in a clinical trial (NCT02663518).

### Small molecules

#### BCL2 inhibitor

**Fig. 6:**
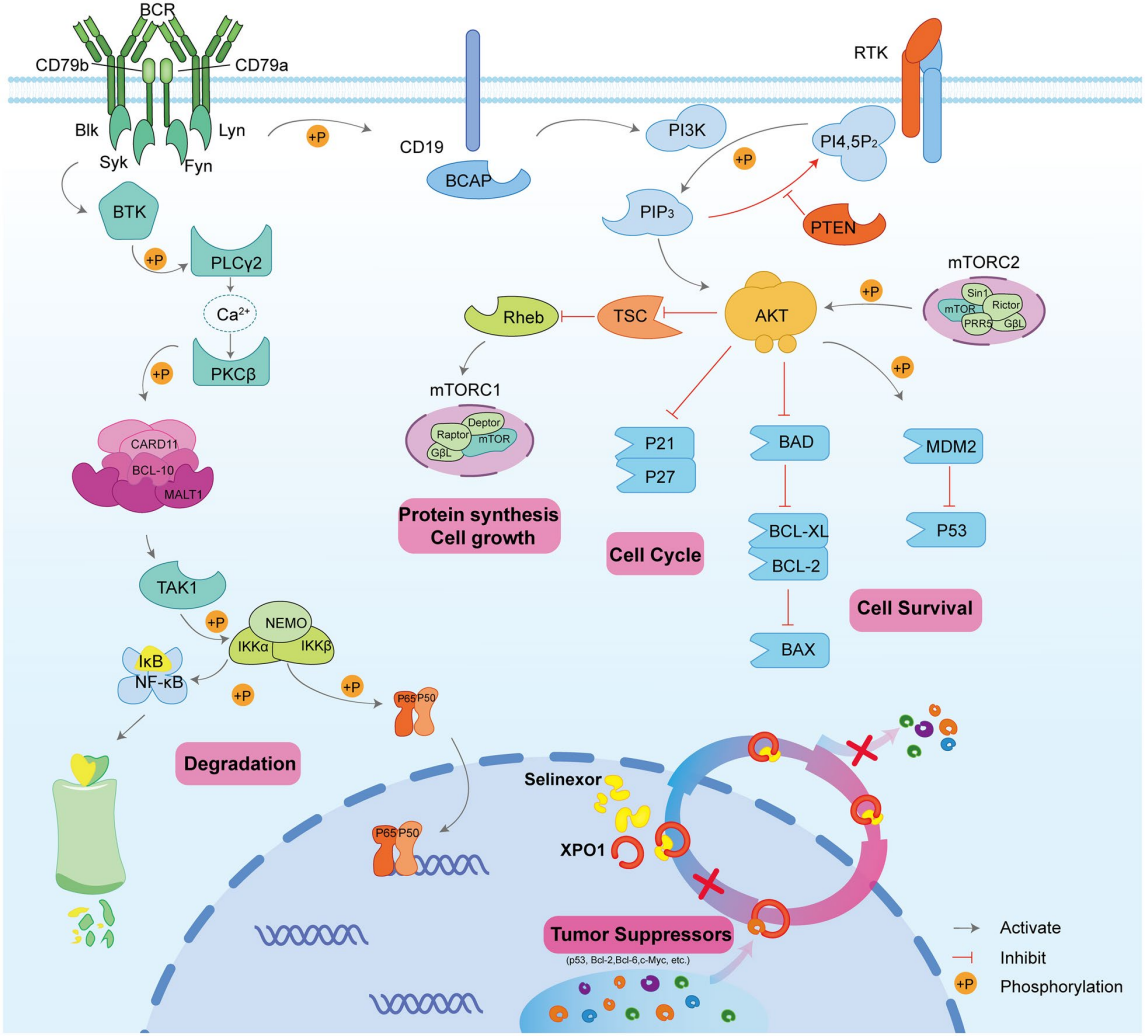
The application of small molecules agents in R/R DLBCLs. Several biomarkers are potentially targeted in R/R DLBCLs, including BCR (PI3K, MTOR), BCL2, XPO1, NF-κB, and CARD11-BCL10-MALT1 inhibitors

BCL2 protein is overexpressed in approximately 30% of DLBCL patients. Venetoclax is a highly selective, potent oral inhibitor of BCL2, which has shown promising clinical efficacy in a range of NHL subtypes (Fig. 6) [153]. In a phase I trial of patients with R/R B-cell NHL, venetoclax showed modest clinical activity in the 34 patients with R/R DLBCL (ORR, 17.6%, with 11.8% of CR) (Table 5) [153]. Most AEs were grade 1–2, and grade 3–4 events were reported in 56% of patients and were dose independent. The most common grade 3–4 hematologic toxicities were anemia (15%), neutropenia (11%), and thrombocytopenia (9%). The incidence of serious AEs was not high (each is less than 3%), mainly hyponatremia, influenza, and lower respiratory tract infection. Based on the moderate clinical efficacy of venetoclax, a group of heavily pre-treated R/R DLBCL patients were treated with venetoclax combined with Pola and rituximab. The investigator-assessed ORR and CR rates were 65% and 38%, respectively, with a median DOR of 5.8 months [31]. The median PFS and OS were 4.4 months and 11.0 months, respectively [31]. Although CAR-T therapy has shown impressive activity among R/R DLBCLs. Only one-third of the patients achieve durable responses, and the rest of them will eventually experience relapse again [60, 62]. Few potential options are available for patients with R/R DLBCL undergoing CAR-T therapy. Recently, Zhu et al*.* reported that venetoclax-based combination therapy resulted in an ORR of 80%, with 30% achieved CR in patients post CAR-T therapy [154]. Ongoing clinical trials are evaluating venetoclax in combination with other agents, including rituximab plus ibrutinib (NCT03136497), obinutuzumab plus lenalidomide (NCT02992522), and R-ICE chemotherapy (NCT03064867) in R/R DLBCLs.

**Table 5:** Novel agents in R/R DLBCLs

Medicine name	Targets	Efficacy	Comments
Single agent			
venetoclax	BCL2	ORR/CRR, 17.6% (6/34)/11.8% (4/34)	NA
Ibrutinib	BTK	ORR/CRR, 25% (20/80)/10% (8/80)	ABC DLBCL: ORR/CRR, 37% (14/38)/16% (6/38) GCB DLBCL: ORR/CRR, 5% (1/20)/5% (1/20)
Zanubrutinib	BTK	ORR/CRR, 29.3% (12/41)/17.1% (7/41)	ABC DLBCL: ORR/CRR, 36% (9/25)/24% (6/25) GCB DLBCL: ORR/CRR, 25% (1/4)/25% (1/4)
Copanlisib	PI3Kα/δ	ORR/CRR, 19.4% (13/67)/7.5% (5/67)	ABC DLBCL: ORR/CRR, 31.6% (6/19)/21.1% (4/19) GCB DLBCL: ORR/CRR, 13.3% (4/30)/3.3% (1/30)
Parsaclisib	PI3Kδ	ORR/CRR, 25% (15/60)/15% (9/60)	BTK inhibitor naïve: ORR/CRR, 25.5% (14/55)/14.5% (8/55) BTK inhibitor experienced: ORR/CRR, 20% (1/5)/20% (1/5)
Temsirolimus	mTORC1	ORR/CRR, 28.1% (9/32)/12.5% (4/32)	NA
Tazemetostat	EZH2	EZH2 mutations: ORR/CRR, 40% (4/10) EZH2 unmutated: ORR/CRR, 17.6% (15/85)	NA
Abexinostat	Pan-HDAC	ORR/CRR, 31.3% (5/16)/6.3% (1/16)	
Mocetinostat	HDAC 1–3, 11	ORR/CRR (110 mg), 26.3% (5/19)/5.3% (1/19)	Total ORR/CRR, 18.9% (7/37)/2.7% (1/19)
Trotabresib	BET	ORR/CRR, 13.0% (3/23)/8.7% (2/23)	
Lenalidomide	IMiDs	ORR/CRR, 27.5% (11/40)15.0 (6/40)	Non-GCB DLBCL: ORR/CRR, 52.9% (9/17)/29.4% (5/17) GCB DLBCL: ORR/CRR, 4.3% (1/23)/4.3% (1/23)
Lenalidomide	IMiDs	ORR/CRR, 27.5% (14/51)/9.8% (5/51)	Non-GCB DLBCL: ORR/CRR, 28.6% (8/28)/14.3% (4/28) GCB DLBCL: ORR/CRR, 26.1% (6/23)/4.3% (1/23) ABC DLBCL: ORR/CRR, 45.5% (5/11)/27.3% (3/11) GCB DLBCL: ORR/CRR, 21.4% (3/14)/7.1% (1/14)
Selinexor	XPO1	ORR/CRR, 28.3% (36/127)/11.8% (15/127)	High/ low Myc protein expression: ORR, 13% (6/47)/42% (22/52) DEL/non-DEL: ORR, 9.7% (3/31)/40.3% (23/57)
Combination			
Ven-OLI		ORR/CRR, 55.6% (15/27)/37.0% (10/27)	Non-GCB DLBCL: ORR/CRR, 61.5% (8/13)/53.8% (7/13) GCB DLBCL: ORR/CRR, 50.0% (7/14)/21.4% (3/14)
CUDC-907-R		ORR/CRR, 63.6% (7/11)/18% (2/11)	MYC non-altered: ORR/CRR, 71.4% (5/7)/ 0% (0/7) MYC-altered: ORR/CRR, 50% (2/4)/50% (2/4)
iR2		ORR/CRR, 49.4% (42/85)/28.2% (24/85)	ABC DLBCL: ORR/CRR, 54.8% (17/31)/32.3% (10/31) GCB DLBCL: ORR/CRR, 35.7% (5/14)/14.3% (2/14)
Temsirolimus-Len		ORR/CRR, 25.6% (10/39)/12.8% (5/39)	Cases of CR: 2 cases of ABC DLBCL, 2 cases of GCB

*R/R* relapsed/refractory, *DLBCL* diffuse large B-cell lymphoma, *BTK* bruton tyrosine kinase, *PI3K* phosphoinositide 3-kinase, *EZH* enhancer of zeste homolog, *HDAC* histone deacetylase, *IMiD* immunomodulatory drug, *XPO1* exportin 1, *DEL* double expressor lymphoma, *Ven-OLI* venetoclax/ibrutinib/lenalidomide/obinutuzumab, *iR2* ibrutinib, lenalidomide, and rituximab, *ORR* overall response rate, *CRR* complete response rate, *ABC* activated B-cell, *GCB* germinal center B-cell, *NA* not applicable

#### BTK inhibitor (BCR signal pathway)

BTK, a crucial component of the B-cell receptor (BCR) signaling pathway, leading to downstream activation of NF-κB, is essential for ABC (or non-GCB) DLBCL cell survival [155] (Fig. 6). Ibrutinib is a first-in-class oral BTK inhibitor for treating B-NHLs [156]. In a phase I/II study, patients with R/R DLBCL demonstrated preferential ORR with ibrutinib in the ABC subtype (37%, 14 of 38 cases) relative to the GCB subtype (5%, 1 of 20 cases) DLBCL (Table 5) [157]. The TEAEs aligned with previous studies, mainly grade 1–2 hematologic toxicities, including anemia, thrombocytopenia, and nonhematological ones. Among these, ABC tumors with BCR mutations responded to ibrutinib frequently (5/9; 55.5%), especially those with concurrent MYD88 mutations (4/5; 80%) [157].

In recent years, more-highly selective BTK inhibitors (such as zanubrutinib and acalabrutinib), in hopes of minimizing ibrutinib’s off-target effects and toxicities (namely bleeding and atrial fibrillation), have shown their clinical efficacies in B cell NHLs, including DLBCLs [158]. In the BGB-3111-207 study, R/R non-GCB DLBCL patients treated with zanubrutinib achieved an ORR of 29.3%, with 17.1% of patients achieving CR. Median DOR, PFS, and OS were 4.5, 2.8, and 8.4 months (Table 5). Grade ≥ 3 AEs in 48.8% of patients, and AEs leading to treatment discontinuation were reported in 4 patients. Bleeding, atrial fibrillation or flutter were not observed [159]. Like ibrutinib, later studies will focus on developing mechanism-based or biomarker-driven treatment combinations (NCT04705129, NCT04460248).

#### PI3K/AKT/mTOR inhibitors (BCR signal pathway)

Class I phosphoinositide 3-kinase (PI3K) comprise four isoforms: PI3Kα, PI3Kβ, PI3Kγ, and PI3Kδ. The PI3K signaling pathway has been activated in numerous human malignancies, including indolent NHLs and aggressive NHLs [160]. Aberrant PI3K/AKT/mTOR pathway activation is observed in a significant subset of DLBCL samples and is driven by chronic activated BCR signaling [10–13] (Fig. 6). Although PI3Kδ inhibitor idelalisib showed only modest activity in R/R DLBCLs [161], combined inhibition of the PI3Kα/δ isoforms shows promising results, especially in treating ABC DLBCL patients [160]. Patients with R/R DLBCL received copanlisib (PI3Kα/δ inhibitor) as monotherapy in a phase II trial. The ORR was 19.4% in the total cohort but much higher in the ABC group than in the GCB group (31.6% vs. 13.3%) (Table 5) [162]. SAEs occurred in 65.7% of patients (44/67). The most common drug-related TEAEs were hypertension (34.3%), hyperglycemia (31.3%), nausea (17.9%), fatigue (17.9%) and diarrhea (16.4%). Due to the serious side effects of copanlisib, in a phrase II study, parsaclisib, a highly selective, next-generation PI3Kδ inhibitor, was evaluated in patients with R/R DLBCL and showed manageable toxicity. The ORR were 25.5% (14/55) and 20% (1/5) in BTK inhibitor naïve and experienced cases, respectively. Due to the modest efficacy and tolerance, it is reasonable to combine standard therapies and other agents of synergistic with parsaclisib in DLBCLs. Clinical trials are underway investigating parsaclisib in combination with tafasitamab (NCT04661007) and Pola-R-CHOP (NCT04323956).

mTOR comprises two distinct multiprotein complexes, mTORC1 and mTORC2, which contain different proteins and share two subunits. Subunits unique to mTORC1 and mTORC2 are raptor and rictor, respectively (Fig. 6) [163]. Rapamycin analogues (mTORC1 inhibitors), everolimus and temsirolimus (plus rituximab) displayed an ORR of 28–38% with median DOR of 2.4–8.1 months in R/R DLBCLs (Table 5) [164, 165]. A phase II study investigated the safety and efficacy of the dual mTORC1/2 inhibitor vistusertib with or without rituximab in R/R DLBCLs. However, vistusertib did not confer an advantage over mTORC1 inhibitors. The STORM study evaluated the safety and activity of temsirolimus plus R-DHAP as salvage therapy for patients with R/R DLBCL [166]. This treatment was well tolerated, and the most common AEs were leukopenia (75%), thrombocytopenia (83%), anemia (57%), and hypokalemia (25%) [166]. At the end of the last follow-up, The ORR after the last cycle was 72% (36/50) with 42% (12/50) of CR. The median DOR was not reached, and the 2-year PFS and OS were 53% and 59%. Patients who received auto-SCT achieved an ORR of 91% and a CR rate of 65% [166]. Everolimus combined with R-CHOP induced a high CR rate (96%, 23/24) in a phase-II trial of untreated DLBCL patients [167]. The most common grade 3–4 toxicity was neutropenia, with 75% of grade 4 and 21% of patients having grade 3 febrile neutropenia.

#### Targeting CARD11-BCL10-MALT1 (CBM) complex (BCR signal pathway)

MALT1, a key effector of the CBM complex, activates canonical NF-κB and induces the growth of ABC DLBCL. CBM complex is a central effector of the BCR pathway and plays a critical role in NF-κB activation (Fig. 6) [168]. MALT1 regulated metabolism in lymphocytes by promoting the expression of Glutaminase-1 [169]. Several MALT1 protease inhibitors have been investigated in ABC DLBCLs in preclinical models and showed remarkable inhibition on lymphoma cell growth, which could overcome resistance to BTK inhibitors [170]. In addition, CARD11 and BCL10 mutations represent a vital resistance mechanism to BTK inhibitors [171]. Dual blockade of the BCR-CBM-NF-κB pathway with a MALT1 inhibitor led to synergistic suppression of ABC-DLBCL cells [170].

#### Proteasomal inhibitors

Carfilzomib is a potent, tetrapeptide ketoepoxide-based inhibitor first approved for treating R/R multiple myeloma [172]. It could upregulate pro-apoptotic proteins dose- and time-dependently and overcome resistance to chemotherapeutic agents in preclinical R/R DLBCL models [173]. In a prospective phase I study (NCT01959698), R/R DLBCL patients were treated with carfilzomib plus RICE as salvage therapy. For patients who underwent auto-SCT, the ORR was 62%, and 48% of cases achieved CR. The median PFS and median OS were 15.2 months, and 22.6 months, respectively. The non-GCB group benefited more from the C-RICE regimen than the GCB group (CR rate: 65% vs. 13%). These results compared favorably to other salvage regimens reported in previous studies [174–176]. Most grade 3–4 AEs were hematological, including thrombocytopenia (72%), anemia (52%), neutropenia (31%), lymphopenia (3%), and febrile neutropenia (10%). No dose-limiting toxicity was noted. Another proteasomal inhibitor, bortezomib, was combined with R-DAEPOCH in R/R DLBCL patients, which showed similar results in the non-GCB group (ORR: 83% vs. 13%; CR rate: 42% vs. 7%) [177]. Thus, it could be concluded that proteasomal inhibitor-based salvage therapy might be one of the potential strategies in non-GCB R/R DLBCLs.

### Epigenetic modification

#### EZH2 inhibitor

Enhancer of zeste homolog two (EZH2), a histone methyltransferase crucial in GC formation, regulates B cell differentiation and promotes cell proliferation [178]. Mutations of EZH2 are a frequent genetic event (21.7%) in GCB DLBCLs [179]. EZH2 represents a histone methyltransferase, and recurrent activating mutations in the encoding gene were reported to enhance proliferation and block further differentiation of GCB cells [180]. Tazemetostat is an EZH2 inhibitor approved for treating patients with R/R FL. In a multicenter phase II trial, 95 R/R DLBCL patients were treated with oral EZH2 inhibitor tazemetostat. The ORR was 40% in patients with EZH2 mutations (N = 10), 18% in patients with wildtype EZH2 (N = 85) (Table 5) [181]. The common TEAEs were thrombocytopenia and dysgeusia. No treatment-related serious AEs were observed. Valemetostat, an EZH1/2 dual inhibitor, is being evaluated in R/R DLBCLs (NCT04842877).

#### HDAC inhibitor

The acetylation of histone is one of the most crucial epigenetic regulations of gene expression, which plays an essential role in the pathogenesis of hematological malignancies [182]. Histone acetylation is controlled by the balance between histone deacetylases (HDACs) and histone acetyltransferases [183]. Histone deacetylases inhibitors (HDACIs) were found to be a novel therapeutic strategy in aggressive lymphomas [184]. According to chemical structures, HDACIs are mainly divided into four categories, including hydroxamic acids, benzamides, cyclic peptides, and aliphatic fatty acids[185]. Single HDACIs have been investigated in R/R DLBCLs, with an ORR of 5.6–40%, a median PFS of 2–3 months, and a median OS of 7–17 months [185, 186]. Abexinostat, an oral pan-HDACI, showed an ORR of 31%, with 6% CR in R/R DLBCLs (Table 5) [187]. Abexinostat is also being evaluated with ibrutinib in patients with R/R DLBCL (NCT03939182). Mocetinostat, a selective HDACI (selectively inhibits HDAC1, 2, 3 and 11), induced an ORR of 26.3% (6/19) with 5.3% (1/19) of CR in R/R DLBCL patients with a dose of 110 mg (Table 5) [188]. The most frequent AEs were fatigue (75.0%), nausea (69.4%) and diarrhea (61.1%) [188]. Several preclinical studies demonstrated that HDACIs potently enhance the anti-tumor activity of rituximab, partially by upregulating CD20 expression and targeting the apoptosis signaling pathway [189, 190]. Chidamide, an oral HDACI (selectively inhibits HDAC1, 2, 3, and 10 activities), was approved by the China FDA in 2015 for treating R/R peripheral T cell lymphoma. In the phase II prospective study, chidamide plus rituximab showed a manageable tolerance, with an ORR of 40% in R/R DLBCLs, a median PFS of 2.6 months, and a median OS of 16.7 months [186]. However, the clinical efficacies were inconsistent with different combinations of HDACIs and R-CHOP [191–193]. Different previous treatment regimens and target categories of HDACs might be reasons for the conflicting results. HDACIs, in combination with drugs targeting CD20, could be one of the future directions of lymphoma therapy. Identifying predictive markers (such as CREBBP/EP300 mutations) of activity might improve the outcomes [194].

#### Bromodomain and extra-terminal inhibitor

Bromodomain and extra-terminal (BET) proteins act as “epigenetic readers” of histone acetylation. They regulate gene expression, cancer-cell proliferation, survival, and oncogenic progression of B-cell NHL, where they might activate the MYC and BCL2 pathways [195, 196]. Monotherapy with BET inhibitors (INCB057643, INCB054329, CC-90010, CPI-0610, birabresib, RO6870810) had discouraging results in R/R DLBCLs (ORR: 0–14%) [197]. For example, trotabresib, an oral BET inhibitor, showed low antitumor activity in R/R DLBCLs, with an ORR of 13.0% (3/23) (Table 5) [198]. However, R/R DLBCL patients who received RO6870810 in combination with venetoclax and rituximab showed promising results [199]. In this phase Ib study, the ORR was 39%, with 21% of patients achieving CR, and 48% had a DOR ≥ 6 months [199]. The most frequent grade 3–4 AEs were neutropenia (28%), anemia (23%) and thrombocytopenia (23%) [199]. Based on these, BET and BCL2 inhibitors may provide therapeutic potential for patients with MYC or/ and BCL2 genes altered DLBCLs.

#### Protein arginine N-methyltransferases (PRMTs) five inhibitor

PRMTs catalyze histone proteins’ arginine methylation, resulting in gene silencing [84]. PRMT5 is highly expressed in EBV-positive human lymphoma and is associated with inferior outcomes [200, 201]. PRMT5 is required to form GCB and interact with MYC, which might be an effective target in patients with MYC-driven GCB DLBCL [201, 202]. The PRMT5 inhibitors, GSK3326595 and JNJ-64619178, are being evaluated in B-NHLs (NCT02783300, NCT03573310).

#### Immunomodulatory drugs

Most novel agents are created and developed to target the tumor cells, such as mAbs, ADCs and BsAbs. Nonmalignant components of the tumor microenvironment, such as T cells, NK cells, tumor-associated macrophages, and dendritic cells, are demonstrated to play essential roles in lymphoma progression and survival, facilitating the ability of malignant B cells to avoid recognition and destruction by the immune system. Compared to the direct anti-tumor effects on malignant B cells and their ability to activate cytolytic immune cells, immunomodulatory drugs (IMiDs), such as lenalidomide, can not only exert their anti-malignant effect by targeting the tumor cells but also modulating several nonmalignant components of the TME and overcoming the immunosuppressive TME, which makes them excellent candidates for combinational immunotherapies. Lenalidomide, initially approved in 2005, is now one of the most frequently used IMiDs of multipotent, either alone or in combination, for hematological malignancies, including DLBCLs [203–207].

In ABC-DLBCL cell lines, lenalidomide exhibits anti-tumor activity via downregulating IRF4 and SPIB transcription factors, leading to cell cycle arrest and apoptosis [208]. In an early retrospective study, lenalidomide demonstrated preferential activity in non-GCB (ABC)-DLBCL (ORR, 52.9%, CR, 23.5%, median PFS, 6.2 months) compared to the GCB subtype (ORR, 8.7%, CR, 4.3%, median PFS, 1.7 months) (Table 5) [209]. In another phase II/III trial, the ORRs were similar between these two groups based on the Hans algorithm. Still, a significant clinical outcome difference was demonstrated (ORR of 45.5% for ABC-DLBCL vs. 21.4% for GCB-DLBCL) based on GEP classification (Table 5) [210]. Unlike lenalidomide, avadomide, a next-generation IMiDs, has shown direct apoptotic activity in DLBCL cells and promising clinical activity in R/R DLBCL patients with both GCB- and ABC-DLBCL subtypes [211]. Thus, IMiDs may be excellent partners of novel agents and traditional chemotherapies in the treatment setting of DLBCLs.

#### XPO1 inhibitor

Exportin 1 (XPO1), overexpressed in DLBCL patients, is a nuclear exporter responsible for exporting proteins that contain a nuclear export signal (NES) out of the nucleus to the cytoplasm [212] (Fig. 6). High XPO1 expression is associated with advanced clinical stages and poorer outcomes in DLBCLs [212, 213]. Selinexor is an oral therapeutic drug that makes a reversible covalent bond with XPO1 and selectively inhibits XPO1-associated nuclear export. In a phase I trial, selinexor was evaluated in 41 patients with R/R DLBCL, and there was an ORR of 32% with 10% cases achieved CR [214]. In the phase IIb SADAL study, 127 patients with R/R DLBCL were treated with selinexor (60 mg). The ORR was 28%, with 12% of CR and 17% of PR (Table 5). With a median follow-up of 14.7 months, the median DOR, PFS and OS were 9.3, 2.6 and 9.1 months, respectively [213]. The ORR was higher in the patients with GCB subtype (ORR, 34% vs. 21%) and low (cutoff, 40%) c-Myc expression (ORR, 42% vs. 13%). Similar result was recognized in the patients with DEL (ORR, 9.7%, 3/31) and without DEL (ORR, 40.3%, 23/57). The most common grade 3–4 AEs were thrombocytopenia (46%), neutropenia (24%), anemia (22%), and fatigue (11%) [213]. Based on these results, selinexor was approved by the FDA for patients with R/R DLBCL after at least two prior systemic therapies. Despite these successes, not all patients respond effectively to XPO1 inhibition, and there has been a lack of biomarkers for response to XPO1 inhibitors in the clinic. Totiger et al*.* identified XPO1, MCL-1, NF-κB and p53 expression as potential predictive biomarkers of response to XPO1 inhibitor therapy [215]. Although R/R DLBCL with c-Myc overexpression showed poor responses to selinexor, combination therapies with other novel drugs may induce responses, such as ibrutinib, fimepinostat (CUDC-907), which had shown promising clinical efficacies [216, 217]. Several clinical trials are evaluating selinexor alone (phase 2b study, NCT02227251) or in combination with novel agents such as venetoclax (NCT03955783), CAR-T cell therapy (NCT05322330) in R/R DLBCLs, and with chemotherapy in both R/R DLBCLs (NCT04442022, NCT05786989) and de novo DLBCLs (NCT05577364, NCT05422066, NCT03147885).

#### Target CD74

CD74, a transmembrane glycoprotein that functions as a survival receptor, is highly expressed on the cell surface of B cells, regardless of clinical stages [218–220] (Figs. 1, 2, 3). Milatuzumab, the first anti-CD74 mAb approved by the FDA for clinical practice, is effective at treating aggressive B cell malignancies, especially in combination with rituximab [219, 221]. STRO-001, a novel anti-CD74 ADC, induced a modest ORR of 25% (4/16) in heavily pre-treated NHL patients, including two DLBCL patients who had previously progressed after CAR-T cell therapy [222]. Geanes et al*.* engineered BiTEs targeting CD74 with rituximab anti-CD20 (anti-CD74/anti-CD20) [220]. The BiTEs caused significantly more apoptosis than anti-CD20 alone in both the rituximab intermediate (NU-DUL-1) and rituximab resistant (SU-DHL-8) cells [220]. BsAbs targeting CD74 and CD20 could mediate antibody-dependent cellular cytotoxicity (ADCC), antibody-dependent cellular phagocytosis and direct cellular cytotoxicity similar to anti-CD20 [220]. These data demonstrate that the dual specificity of engineered BsAbs (including CD20 and CD19 mentioned above) are compelling cancer immunotherapy prospects.

#### Other agents in exploration

Although the median DOR was not reached, Urelumab, a CD137 agonist mAb, combined with rituximab, showed limited clinical activity but increased toxicity in heavily pre-treated DLBCL patients [223]. Further efforts are needed to reduce the toxicity and improve the effectiveness of urelumab. CD37 is highly expressed in malignant B-cells. IMGN529, an ADC comprised of a humanized anti-CD37 mAb linked to DM1, showed a manageable safety profile and encouraged clinical efficacy (ORR, 22.2%) in patients with R/R DLBCL [224]. Apatinib, a new oral kinase inhibitor mainly targeting vascular endothelial growth factor receptor two, showed promising efficacy and manageable toxicities in patients with R/R DLBCL [225]. Further investigations of the combination therapy of apatinib with other novel synergistic agents are reasonable. Napabucasin, a novel STAT3 inhibitor, showed significant synergism with doxorubicin in both vitro and in vivo, which is another promising therapeutic candidate for R/R DLBCL patients [226].

### Combination of molecularly targeted agents

#### BTKi + BCL2i

Although single-agent molecular targets have only modest responses in DLBCL, combining these agents seem to have a unique synergy with much-improved efficacies. One study reported by Zhou et al. showed the combination of ibrutinib and venetoclax in R/R DLBCL patients with non-GCB subtype and BCL2 overexpression. The ORR at two cycles was 61.5% (8/13), with 23.1% (3/13) cases achieved CR [227]. The combination of venetoclax/ibrutinib/lenalidomide/obinutuzumab showed ORR and CR rates of 62% (8/13) and 54% (7/13) in non-GCB and 50% (7/14) and 21% (3/14) in GCB DLBCL, with durable responses seen in heavily pretreated patients including patients who underwent CAR-T cell therapy (Table 5) [228]. In the latest report from Smart Start, the ORR after two cycles of rituximab, lenalidomide, and ibrutinib (RLI) was 86.2%, and the CR rate at the end of RLI-chemotherapy was 94.5% [229].

#### BTKi + PI3Ki

Based on genetic alterations of central components of the BCR or its downstream signaling effectors in some subtypes of DLBCL. Various drug combinations have been investigated in preclinical models. BCR signaling and PI3K cascades have been proposed as potential combinations for treating patients with R/R DLBCL. Inhibition of PI3Kα/δ resulted in tumor regression in an ibrutinib-resistant CD79B^WT^/MYD88^mut^ patient-derived ABC-DLBCL model [160]. The combination of the PI3Kα/δ inhibitor AZD8835 and ibrutinib was highly synergistic and effective in both in vitro and in vivo ABC DLBCL models [230]. Combining ibrutinib with the PI3Kα/δ inhibitor copanlisib produced a sustained CR in vivo in CD79B^mut^/MYD88^mut^ ABC-DLBCL models [160]. Another study verified the synergistic effects of ibrutinib and PI3Kγ inhibitor (AS-605240) in DLBCL cell lines [231].

#### PI3Ki + BCL2i

As shown above, Copanlisib (PI3Kα/δ inhibitor), alone or combined with BTK inhibitor, exhibited synergistic effects in BCR-dependent DLBCLs. It was reported that copanlisib could induce apoptosis by modulating Bcl-xL and Mcl-1, which BCL2 inhibitors might enhance. In BCR-dependent DLBCLs, a study found the synergistic activity of copanlisib and venetoclax in a xenograft model [232]. Duvelisib, another PI3K inhibitor (PI3Kδ/γ inhibitor), could lead to ubiquitination and degradation of both c-Myc and Mcl-1, making lymphoma cells more sensitive to BCL2 inhibitor. In patient-derived xenograft models, dual targeting of PI3K-δ/γ and BCL2 led to CR at the end of treatment [233]. Thus, PI3K inhibitor in combination with BCL2 inhibitor might be one of the potential options for R/R DLBCL patients with relevant genetic alterations.

#### PI3Ki + mTORi

PI3K-AKT-mTOR signaling cascade is known to be deregulated in various cancers and represents a major regulator of cell survival, cell proliferation, and angiogenesis. Aberrant PI3K/AKT/mTOR pathway activation is observed in a significant subset of DLBCL samples and is driven by chronically activated BCR signaling [10–13]. PI3Kβ/δ inhibition could decrease the pro-survival NF-κB and AP-1 activity or lead to downregulating the oncogenic transcription factor MYC [234]. However, feedback activation of the PI3K/AKT/mTOR pathway was indicated in PI3Kβ/δ inhibitor-resistant models [234]. The combined treatment with AZD8186 (PI3Kβ/δ inhibitor) and the AZD2014 (mTOR inhibitor) overcame resistance to PI3Kβ/δ inhibition and ultimately prevented the outgrowth of lymphoma cells both in vivo and vitro [234].

#### HDACi + PI3Ki

Several studies have demonstrated that alterations of *MYC* in DLBCL patients indicate dismal outcomes [235–239]. However, the optimal treatment strategies for patients with *MYC*-altered R/R DLBCL remain poorly defined. HDAC and PI3K inhibitors have been reported to reduce MYC protein expression and have synergistic anti-cancer effects [217]. CUDC-907 (fimepinostat) is a small-molecule inhibitor targeting both HDAC (class I and II) and PI3Ks (class Ia, Ib, and Id), which is more potent than single-targeting HDAC or PI3K inhibitors [217]. Preclinical results have shown that CUDC-907 decreases MYC expression and induces apoptosis in double-hit DLBCL cells [240]. In the phase I trial, 37 patients with R/R DLBCL received CUDC-907 with or without rituximab [217]. The ORR was 30% (11/37), with 47% (9/19) in the monotherapy group and 18% (2/11) in the group of combination therapy. The ORR in *MYC*-altered DLBCL patients was 64% (7/11), with 71% (5/7) in CUDC-907 monotherapy and 50% (2/4) in the combination of CUDC-907 and rituximab [217]. However, in *MYC* non-altered patients, the ORR was only 29% (2/7) and 17% (2/12) in those with unknown *MYC* status (Table 5) [217]. The median DOR and median PFS were 11 months and 2.9 months, with 13.6 months and 21.8 months in *MYC*-altered DLBCL patients, six months and 1.3 months in *MYC* unaltered, 7.8 months and 1.3 months in those with unknown *MYC* status [217]. The efficacy and safety of CUDC-907 in patients with *MYC*-altered R/R DLBCL were further evaluated in another phrase II study (NCT02674750) [241].

#### EZH2i and BCL2i

EZB (based on EZH2 mutations and BCL2 translocations) subgroup is one of the genetic subtypes identified by Schmitz et al., which predicted more favorable outcomes than the MCD and N1 subtypes [242]. However, EZH2 and BCL2 protein coexpression was associated with shorter OS and PFS in all DLBCL patients [243]. The combination of EZH2 inhibitor (tazemetostat) and BCL2 inhibitor (venetoclax) showed synergistic effects both in vitro and vivo [244]. Based on these, the combination of EZH2 inhibitor and BCL2 inhibitor might be a potential choice for R/R DLBCL patients with EZH2 mutation and BCL2 gene alterations.

#### Combinations with IMiDs

Preclinical models indicated the potential for synergy with ibrutinib and lenalidomide in ABC DLBCL by inhibiting BCR and MYD88 pathways via distinct mechanisms [245]. In phase 2, patients with R/R non-GCB DLBCL received the combination of ibrutinib, lenalidomide, and rituximab (iR2 regimen). The best ORR was 49% (42/85), with a CR rate of 28% (24/85) [246]. Subgroup analysis by the COO showed the best ORRs were 55% in ABC, 36% in GCB, and 61% in unclassified (Table 5) [246]. Indirect comparisons suggest lower response rates but favorable DOR and OS with the iR2 regimen relative to CAR-T. In particular, the median DOR of 38.3 months and median OS of 12.4 months with the iR2 regimen compares favorably to other novel approved therapies. Because half of the patients receiving CAR-T or auto-SCT treatment would relapse, iR2 may provide a tolerable regimen for individuals who relapsed after or are not candidates for auto-ASCT or CAR-T therapy.

Tumor cells can lead to progressive immune suppression and reflect an immunosuppressed/exhausted phenotype [247]. Components of the TME can also facilitate the ability of malignant B cells to avoid recognition and destruction by the immune system [247]. Treatment with IMiDs can lead to increased T cell activation and proliferation and downregulate the exhaustion-associated marker PD-1 [248]. Lenalidomide can downregulate the expression of immune checkpoint molecules PD-L1 in lymphoma and increase NK cell proliferation and activation via downregulating the expression of immune checkpoint molecule PD-1 on NK cells [249]. Besides, avadomide can upregulate the expression of PD-L1 in the immune TME, which is associated with ‘hot’ inflammatory tumors and sensitive to anti-PD-1/PD-L1 therapies [248]. Thus, combinations of IMiDs and ICIs (anti-PD-1/PD-L1) are alternative therapies worthy of investigation for R/R NHL patients (NCT05058755, NCT05182957, NCT03015896), including R/R DLBCL.

Lenalidomide has been shown to increase NK cell proliferation and activation and augment ADCC. The combination of lenalidomide plus rituximab (R^2^) exhibited enhanced anti-tumor activity in several B-cell NHL patients regardless of front-line or R/R settings, especially in indolent lymphomas [250–255]. Lenalidomide, combined with R-CHOP as front-line therapy, showed promising ORRs and PFS in both FL and DLBCL [256, 257].

The mTOR inhibitor and the immunomodulatory agent have overlapping effects within the PI3K/AKT/mTOR axis with synergistic potential. For combination therapies, temsirolimus and lenalidomide induced an ORR of 25.6% (10/39) with 12.8% (5/39) of CR in R/R DLBCL patients. These findings might be related to cell-of-origin; most responders (7/10), including CR (3/5), harbored an ABC phenotype [258].

Given the established potent activity of several molecular targeted therapies, such as mAbs (NCT05429268), BsAbs (NCT04246086, NCT04663347), BTK (NCT04436107), PI3K (idelalisib) [259], and proteasome (NCT01415752) inhibitors, CAR-T cell therapy (NCT03310619), the potential synergistic effect of these drugs when paired with IMiDs could further improve survival outcomes and efficacy.

#### Auto-SCT

Although auto-SCT is the standard-of-care curative treatment for R/R DLBCL patients who achieve CR after salvage chemotherapy, the relapse rate is usually high, with 50% of patients eventually relapsing [260, 261]. For patients that relapse after auto-SCT within 12 months, only 26% of patients respond to salvage chemotherapy, and the median survival is only 6.3 months [4]. Some R/R DLBCL patients received allo-SCT. However, allo-SCT could induce a high incidence of non-relapse mortality and graft-versus-host disease. CAR-T is a new immune therapy potentially curative for R/R DLBCLs. ZUMA-7 and TRANSFORM trials demonstrated improved EFS for the R/R DLBCL patients with early relapse who after that received CAR-T therapy compared to those treated with SOC [66, 67]. In this alternative setting, some candidates for auto-SCT might transfer to CAR-T therapy. A recent study compared the efficacy of CAR-T and auto-SCT in early relapse DLBCL patients who achieved a PR after salvage chemotherapy. The patients in the auto-SCT group showed better two-year PFS and OS than CAR-T groups [262]. Another small sample study reported improved survival of 14 R/R DLBCL patients treated with auto-SCT and CAR-T therapy. With a median follow-up of 10.3 months, the ORR was 78.6% with no severe toxic effects. The median PFS and OS were 14.8 months and not reached, respectively [263]. Sequential therapy with auto-SCT and CAR-T therapy might be a new pattern for R/R DLBCL patients. Armand et al*.* reported that a negative PETCT scan after savage chemotherapy predicted an improved 4-year PFS in patients who, after that, went for transplant. Besides, in multivariate analysis, including positive PETCT after salvage, symptomatic relapse, and age > 60 were the unfavorable predictors (one point for each factor) of PFS in R/R DLBCLs who undergo auto-SCT [264]. Patients with a high score (3 points) had a 4-year PFS of 0%, while patients with a low score (0–1 point) had a 4-year PFS of 67% [264]. Biologic factors, such as *MYC* gene translocation (or c-Myc protein expression ≥ 40% by immunohistochemistry), will not benefit from auto-SCT [264]. However, auto-SCT is worth trying with DEL [264]. Thus, R/R DLBCL patients with negative predictive factors on auto-SCT should choose other savage strategies, such as CAR-T therapy and novel agents (mentioned above). According to the recommendations from European Bone Marrow Transplantation Society (EMBT), CAR-T cell therapy is now the standard of care for high-risk R/R DLBCL patients who relapse early (chemotherapy insensitive or unknown). In late relapse of DLBCL (chemotherapy sensitive) after standard immunochemotherapy, auto-HCT remains standard of care, although CAR-T therapy could also be considered for these patients [265].

#### Allo-SCT

In the modern era, R/R DLBCL patients can potentially benefit from several approved agents (mentioned above), but these options are generally not expected to provide durable disease control. Cellular immunotherapies directed against defined lymphoma-specific antigens (anti-CD19 CAR-T treatment) or against undefined tumor antigens (allo-SCT, using the graft-versus lymphoma effect) are potentially curative in DLBCLs, even after the failure of high-dose therapy and auto-SCT. Allo-SCT is a potential option for patients with R/R DLBCL but is mainly reserved for medically fit patients with disease progression after auto-SCT or CAR-T cell therapy.

A retrospective study reported 50–60% long-term survival after allo-SCT, but this therapeutic modality has a 40–50% treatment-related mortality [266]. Retrospective analysis of a small sample of patients with DHL/THL who underwent allo-SCT showed similar outcomes (PFS, OS) to those who did not have DHL/THL [267]. In another retrospective analysis using the CIBMTR registry, Hamadani et al*.* describe the outcomes of patients with DLBCL relapsing after auto-SCT and undergoing allo-HCT or CAR-T therapy [268]. The 1-year relapse, non-relapse mortality, OS, and PFS for the allo-SCT cohort after auto-SCT failure were 26.2%, 20.0%, 65.6%, and 53.8%, respectively. The corresponding rates in the CAR-T treatment were 39.5%, 4.8%, 73.4%, and 55.7%, respectively. The 1-year OS of allo-SCT recipients was classified as low (73.3%), intermediate (59.9%) and high/very high-risk (46.3%) groups according to the CIBMTR prognostic score. The corresponding rates for low-, intermediate-, and high/very high-risk CAR-T patients were 88.4%, 76.4%, and 52.8%, respectively (*P* < 0.001) [268].

In the recent EMBT guidelines, the role of allo-SCT was modified. Allo-SCT is considered only an option. However, for patients with DLBCL failure after auto-SCT, allo-SCT and CAR-T are available options depending on patients’ characteristics and access to medication. It is generally believed patients who are younger, fit, sensitive to salvage treatments, and carrying a high tumor burden are more suitable for allo-SCT. In contrast, those who are older, unfit, and refractory to prior lines of regimens should better receive CAR-T therapy. However, most patients will fail CAR-T therapy, resulting in unmet medical needs where allo-SCT could be beneficial.

In contrast, employing allo-HCT instead of CAR-T therapy as the first choice should be restricted to situations where CAR-T therapy is deemed unfeasible or valuable [269]. An expert panel opinion from the American Society for Transplantation and Cellular Therapy suggested that allo-SCT may be considered for selected patients in CR after CAR-T cell therapy under individualized evaluation. In contrast, in patients with relapse/progression, allo-SCT should be included among the treatment options [270]. Because of the toxicity, allo-SCT should only be considered in a few selected patients, such as patients with stable disease (SD) after CAR-T cell therapy [271]. For selected patients with CR and SD, identifying risk factors to predict who may relapse or progress sooner may be beneficial in deciding which patients should proceed to allo-SCT, especially in high/very high-risk groups who failed CAR-T cell therapy according to the CIBMTR prognostic score [268, 272]. In all, for patients failing second-line therapies, relapsing after auto-SCT or with refractory disease, allo-SCT remains a clinical option after failure of CAR-T therapy.

### Radiotherapy

NCCN guidelines recommend radiotherapy (RT) for DLBCL patients with early-stage or advance-stage with or without the bulky disease who show a residual disease at end-of-treatment. However, whether RT should be used as consolidative therapy after inductive treatment in either early or advanced disease remains controversial [273]. In patients with R/R DLBCL, high-dose chemotherapy followed by auto-SCT has become the standard of care for eligible patients [1]. However, half of the patients who underwent auto-SCT still experience recurrence, and there has been limited standard salvage chemotherapy [4]. The rationale of RT for selecting R/R DLBCLs as a part of the salvage program is mainly based on the high incidence of local recurrence, which has become a significant problem, despite an excellent response to salvage therapy [274]. About 40% and 76% of early-stage and advanced-stage patients had developed a relapse at the sites of origin, which indicates local disease control is of great importance [275]. Although DLBCL is a systemic disease, patients with localized relapses who undergo RT contribute to durable responses and favorable outcomes [276]. Roles of RT in R/R DLBCLs include consolidative therapy for auto-SCT eligible patients or palliative intent for frail ones [277]. Besides, patients with dominant skeletal relapses showed a 70% improvement in disease control after RT [278]. Most studies showed that receiving RT was associated with improved outcomes regardless of pre- or post-transplant RT [274], and RT does not preclude or diminish the efficacy of subsequent therapies [276]. However, both pre- and post-transplant RT has their advantages and disadvantages, which depend on the disease state and patients’ characteristics. In transplant-ineligible patients, RT can provide effective palliation or curative results for localized disease. RT should be strongly considered for life-threatening sites where local control is especially critical. Patients with chemotherapy-resistant R/R DLBCLs often demonstrate RT sensitivity [279].

CAR-T cell therapy requires a long manufacturing period for disease control or alleviating symptoms, so bridging therapy may be necessary [279]. RT has been considered an effective bridging therapy to meet that unmet need to stabilize the disease and keep patients fit until the successful infusion of CAR-T cells [279]. The rationality of using RT as a bridge therapy before CAR-T cell therapy lies in the potential synergistic effect on the vitality of CAR-T cells [280]. As mentioned, salvage RT as bridge therapy before CAR-T cell infusion had promising clinical efficacy for patients with PR or local relapse (low tumor burden). Besides, RT can also be used as salvage therapy of R/R DLBCLs following CAR-T cell therapy [281, 282]. Due to its low toxicity and ease of use, pre- or post-RT should remain available in patients with R/R DLBCL. However, several questions remained unacknowledged in optimizing salvage RT for R/R DLBCL patients who have undergone or will receive CAR-T cell therapy. Several prospective clinical trials are under investigation to answer these questions (NCT04473937, NCT04790747, NCT04601831).

## Further directions and conclusions

**Fig. 7:**
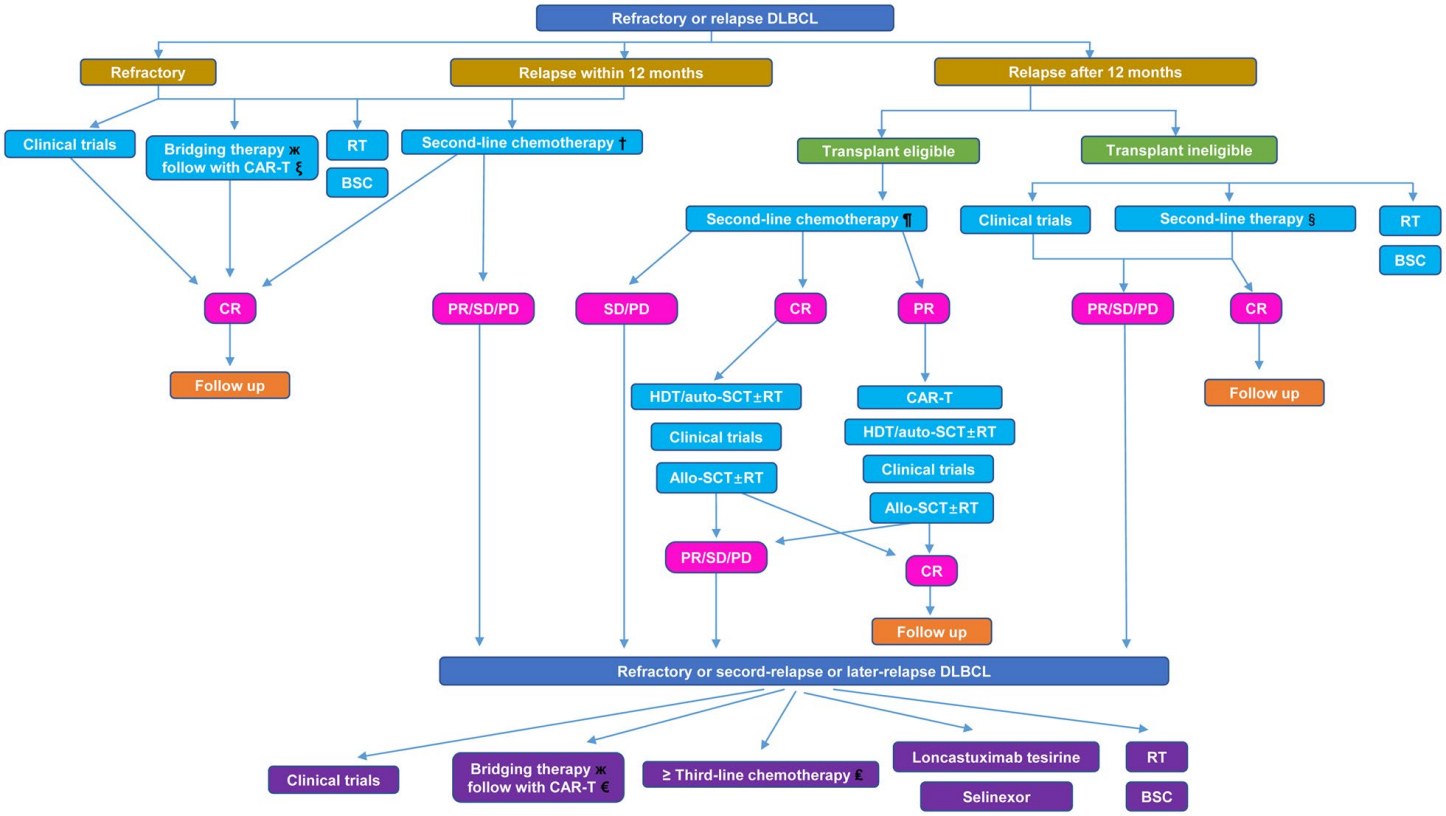
The recommended therapies for R/R DLBCLs. This chat shows the recommended therapies with patients of R/R DLBCL in different clinical states. *DLBCL* diffuse large B-cell lymphoma, *CAR-T* chimeric antigen receptor T cells, *RT* radiotherapy, *BSC* best supportive care, *CR* complete response, *PR* partial response, *SD* stable disease, *PD* progressive disease, *HDT* high-dose chemotherapy, *SCT*, stem cell transplantation. ¶ second-line chemotherapy for transplant eligible: DHAP ± R, GDP ± R, ICE ± R, ESHAP ± R, GemOx ± R, MINE ± R, § second-line therapy for transplant ineligible: CAR-T (Liso-cel), Pola ± B ± R, Tafa + Len, CEOP ± R, DA-EPOCH ± R, GDP ± R, GemOx ± R, Rituximab, BV, BTKi, Len ± R, † second-line chemotherapy for relapse within 12 months or refractory disease: alternative systemic therapy, Ж, Bridging therapy: DHAP ± R, GDP ± R, ICE ± R, Pola ± B ± R, RT, ξ CAR-T products: Axi-cel, Liso-cel, € CAR-T products: Axi-cel, Liso-cel, Tisa-cel, ₤ ≥ Third-line chemotherapy: alternative systemic therapy

Although two-thirds of patients with DLBCL can be cured with standard first-line immunochemotherapy, one-third remain refractory to initial treatment or relapse after the first remission. For over a decade, high-dose salvage chemotherapy followed by auto-SCT remained the standard for fit and chemotherapy-sensitive patients. However, only half of these patients failed to benefit from this strategy. In recent years, CAR-T cell therapy provided durable responses in a proportion of patients and has changed the treatment landscape of R/R DLBCLs. For patients of early relapse or primary refractory disease, Axi-cel and Liso-cel are now recommended second-line therapies by NCCN guidelines. For those unsuitable for auto-SCT or CAR-T therapy, Pola-BR or tafasitamab in combination with lenalidomide is also recommended (Fig. 7). Other candidate choices, including BV for CD30-positive cases, ibrutinib and lenalidomide with or without rituximab for the non-GCB group, are also helpful therapies (Fig. 7). Many novel agents like selinexor and Lonca, including CAR-T cell therapies, are recommended for third and later-line therapy (Fig. 7).

The problem of post-CAR T-cell relapse and patients’ refractory to CAR T-cell therapy is particularly challenging. BsAbs showed promising efficacy in CAR-T naïve and refractory patients with more manageable safety profiles and off-the-shelf than CAR-T therapy. ICIs, being assessed by clinicians, and other targeted approaches are needed to overcome or reverse this awkward situation. Many new agents targeting apoptosis (BCL2 inhibitor), BCR signal pathway (BTK, PI3K, mTOR inhibitors and so on), epigenetics, and TME, though of limited benefit, are emerging. Clinical trials continue exploring the efficacy and safety of various drug combinations (Table 6). Thus, we urgently need biomarkers that can predict the effectiveness of these novel drugs.

**Table 6:** Available options after CAR-T cell therapy failures in R/R DLBCLs

Treatments	Targets	Efficacy in R/R DLBCLs	Post CAR-T efficacy	Common toxicities
Pola-BR (GO29365) NCT02257567	Anti-CD79b/anti-CD20 immunochemotherapy	ORR/CRR, 70% (28/40)/57.5% (23/40) mDOR, 10.3mo mPFS, 7.6 mo mOS, 12.4 mo [29]	ORR/CRR, 72%/33% [283]	Anemia (53.8%), neutropenia (53.8%), thrombocytopenia (48.7%), diarrhea (38.5%), nausea (30.8%), fatigue (35.9%), pyrexia (33.3%), peripheral neuropathy (43.6%)
Loncastuximab-tesirine	Anti-CD19 immunotherapy	ORR/CRR, 48.3% (70/145)/24.1% (35/145) mDOR, 10.3 mo mPFS, 4.9 mo mOS, 9.9 mo [19]	ORR/CRR, 46.2% (6/13)/15.4% (2/13) Median DOR, 8 mo Median PFS, 1.4 mo Median OS, 8.2 mo [284]	Mild (no adverse events occurred more than 30%)
Tafasitamab-lenalidomide	Anti-CD19 immunotherapy/ immunomodulation	ORR/CRR 57.5% (46/80)/ 40% (32/80) mDOR, 43.9 mo mPFS, 11.6 mo mOS, 33.5 mo [12]	ORR/CRR, 33%/17% [283]	Neutropenia (49%), anemia (34%), thrombocytopenia (31%), rash (36%), diarrhea (33%)
Mosenetuzumab (NCT02500407)	T-cell-engaging bispecific antibody	ORR/CRR, 33% (13/39)/ 21% (8/39) [49]	ORR/CRR, 36.8% (7/19)/26.3% (5/19) [50]	Neutropenia (28.4%), cytokine release syndrome (27.4%), hypophosphatemia (23.4%), fatigue (22.8%), diarrhea (21.8%)
Glofitamab (NCT03075696)	T-cell-engaging bispecific antibody	ORR/CRR, 51.6 (80/155)/39.4% (61/155) [57]	not available	Pyrexia (46.2%), hypotension (24.6%), tachycardia (15.8%), chills (12.3%)
Odronextamab ≥ 80 mg (NCT02290951)	T-cell-engaging bispecific antibody	ORR/CRR, 60% (6/10)/60% (6/10) [39]	ORR/CRR, 33.3% (7/21)/23.8% (5/21) mDOR, 2.4 mo [39]	pyrexia (76.4%), CRS (62.2%), and chills (48.0%)
Epcoritamab (NCT03625037)	T-cell-engaging bispecific antibody	ORR/CRR, 63.1% (99/157)/38.9% (61/157) mDOR, 12.0 mo mPFS, 4.4 mo mOS, not reached [54]	ORR/CRR, 54% (33/61)/34% (21/61) [54]	CRS (49.7%), injection site reaction (19.7%), and neutropenia (17.8%)
Selinexor (NCT02227251)	XPO1 inhibitor	ORR/CRR, 28.3% (36/127)/11.8% (15/127) mPFS 2.6 months mOS 9.1 months [213]	Not available	Thrombocytopenia (61%), nausea (58%), fatigue (47%), anaemia (43%), decreased appetite (37%), diarrhoea (35%), constipation (31%), neutropenia (30%), weight loss (30%)
Allo-SCT	Allogeneic hematopoietic stem cell transplantation	ORR/CRR, 73.9% (17/23)/69.6% (16/23) [266]	Not available	cGVHD at 1 year (48%)
Pembrolizumab (49%) Nivolumab (43%) Atezolizumab (6%) Others (2%)	PD-1/PD-L1	ORR/CRR, 11.8%/7.8% mDOR, 7.5 mo mPFS, 1.8 mo mOS, 4.7 mo [79]	ORR, 33–35% [283]	Cytopenias, infections, pneumonitis, colitis, and hepatotoxicity (no adverse events occurred more than 30%)
Ibrutinib based	BTK inhibitor	ORR (ABC group), 37% (14/38) ORR (GCB group), 5% (1/20) [157]	ORR/CRR, 38%/25% [283]	Fatigue (40%), diarrhea (38%), nausea (30%)
Lenalidomide based	Immunomodulation ± immunochemotherapy	ORR/CRR (R2), 28% (9/32)/22% (7/32) [255]	ORR/CRR, 58%/29% [283]	Anemia (88%), neutropenia (100%), thrombocytopenia (94%), leukopenia (91%), lymphopenia (100%), non-neutropenic fever (42%), fatigue (83%), constipation (38%), nausea (43%), neuropathy (58%), blurred vision (31%), myalgia (49%), diarrhea (51%), elevated liver function test (35%), hypocalcemia (30%), raised lactic dehydrogenase (53%), hyperglycemia (47%), hypoalbuminemia (40%), hypophosphatemia (36%)
R-ICE/R-DHAP	Immunochemotherapy	ORR/CRR, 63%/38% [174]	ORR/CRR, 35%/12% [283]	Grade 3/4 hematologic toxicities were more severe in the R-DHAP, more patients required at least one platelet transfusion during the induction phase (57% in R-DHAP arm v 35% in R-ICE arm)
Local radiotherapy	Salvage radiotherapy	ORR/CRR, 78.5% (11/14) /57.1% (8/14) [276]	ORR/CRR, 25%/13% [283]	Not available

*CAR-T* chimeric antigen receptor T cell therapy, *R/R* relapsed/refractory, *DLBCL* diffuse large B-cell lymphoma, *Pola-BR* Polatuzumab vedotin—bendamustine + rituximab, *ORR* overall response rate, *CRR* complete response rate, *mDOR* median duration of response, *mOS* median overall survival, *mPFS* median progression-free survival, *mo* months, *CRS* cytokine release syndrome, *XPO* exportin, *ABC* activated B-cell, *GCB* germinal center B-cell, *BTK* bruton tyrosine kinase

Meanwhile, the combinations of novel agents with traditional therapies, such as RT and chemotherapies, should not be neglected (Fig. 7). Although with a limited application range, allo-SCT is potentially curative, especially in high-risk groups who failed CAR-T cell therapy. There is an unmet need for improved treatment alternatives in frail patients with R/R DLBCL who are ineligible for intensive chemotherapy or CAR-T cell therapy (Fig. 7). Specific mutations have been suggested to define novel molecular subtypes associated with distinct pathogenic mechanisms in DLBCL. These findings may enable the identification of future rational targeted therapies. However, due to the tremendous genetic heterogeneity of DLBCL, there is still a long way to go to achieve precise and personalized treatment in patients with DLBCL.

## Data Availability

Not applicable.

## Abbreviations

ABC: Activated B-cell
ADCC: Antibody-dependent cell mediated cytotoxicity
ADCP: Antibody-dependent cell-mediated phagocytosis
ADCs: Antibody–drug conjugates
AEs: Adverse events
allo-SCT: Allogeneic stem cell transplant
Atezo: Atezolizumab
auto-SCT: Autologous stem cell transplantation
Axi-cel: Axicabtagene ciloleucel
BCR: B-cell receptor
BET: Bromodomain and extra-terminal
BR: Bendamustine + rituximab
BsAbs: Bispecific antibodies
BTK: Bruton tyrosine kinase
BV: Brentuximab vedotin
CAR-T: Chimeric antigen receptor T cell therapy
CDC: Complement dependent cytotoxicity
cHL: Classic Hodgkin's lymphoma
CI: Confidence interval
CR: Complete response
CRR: Complete response rate
DEL: Double expressor lymphoma
DHL: Double-hit lymphoma
DLBCL: Diffuse large B-cell lymphoma
DOR: Duration of response
EBV: Epstein barr virus
EZH2: Enhancer of zeste homolog two
FCR: Fc receptor
FL: Follicular lymphoma
Len: Lenalidomide
R2: Rituximab + lenalidomide
FL3B: Follicular lymphoma grade 3B
FLIC: First-line immunochemotherapy
GA101: Obinutuzumab
GCB: Germinal center B-cell
G-CHOP: GA101 plus CHOP
GemOx: Rituximab, gemcitabine, and oxaliplatin
Gpt: Obinutuzumab
HDACIs: Histone deacetylases inhibitors
HDACs: Histone deacetylases
HGBCL: High-grade B-cell lymphoma
HR: Hazard ratio
ICIs: Immune checkpoint inhibitors
IMiDs: Immunomodulatory drugs
iNHL: Indolent non-Hodgkin lymphoma
INV: Investigator
IV: Intravenous
Liso-cel: Lisocabtagene maraleucel
Lonca: Loncastuximab tesirine
mAbs: Monoclonal antibodies
MMAE: Monomethyl auristatin
mo: Months
NEs: Neurotoxicity events
NES: Nuclear export signal
NHL: Non-Hodgkin lymphoma
NOS: Not otherwise specified
NR: Not reached
ORR: Overall response rate
OS: Overall survival
PFS: Progression-free survival
PI3K: Phosphoinositide 3-kinase
Pina: Pinatuzumab vedotin
PMBCL: Primary mediastinal large B-cell lymphoma
Pola: Polatuzumab vedotin
PR: Partial response
PRMTs: Protein arginine N-methyltransferases
R/R: Relapsed or refractory
R-GemOx: Rituximab + gemcitabine + oxaliplatin
sALCL: Systemic anaplastic large-cell lymphoma
SAR3419: Coltuximab ravtansine
SC: Subcutaneous
SOC: Standard of care
TAA: Tumor associated antigen
TCR: T cell receptor
tFL: Large-cell transformation from follicular lymphoma
THL: Triple-hit lymphoma
THRBCL: T-cell– or histiocyte–rich large B-cell lymphoma
TILs: Tumor-infiltrating lymphocytes
Tisa-cel: Tisagenlecleucel
TMEs: Tumor microenvironments
TTNT: Time to next treatment
Ven: Venetoclax
VH: Heavy chain variable region
VL: Light chain variable region
XPO1: Exportin 1
